# Supplementary material for: Antithrombotic therapy for secondary prevention in patients with stroke or transient ischemic attack: A multiple treatment network meta-analysis of randomized controlled trials
Source: PLoS One. 2022 Aug 17;17(8):e0273103. doi: 10.1371/journal.pone.0273103 (PMC9385057; doi:10.1371/journal.pone.0273103)
Supplement: S1 File — The legends of the included materials can be found in the supplemental material file. (DOCX) [file pone.0273103.s002.docx]

**Supplemental material**

**Antithrombotic therapy for secondary prevention in patients with stroke or transient ischemic attack: a multiple treatment network meta-analysis of randomized controlled trials**

**Table of contents**

[S1 Figure: Visual estimation of publication bias with funnel plot for stroke events: 2](#_Toc107441615)

[S2/A Figure: Bias assessment graph: 3](#_Toc107441616)

[S2/B Figure: Bias assessment summary report: 4](#_Toc107441617)

[S3 Figure: Supplement heat plot of each intervention: 5](#_Toc107441618)

[S4 A/B Figures: Analysis of disability outcome: 6](#_Toc107441619)

[S1 Table: Characteristics of included trials in our meta-analysis: 7](#_Toc107441620)

[S2 Table: Clinical characteristics of the included patient populations: 23](#_Toc107441621)

[S3 A/B Tables: Summary tables of the GRADEpro evaluation of the included cilostazol studies: 28](#_Toc107441622)

[S4 Table: Individual and network meta-analysis results of the risk of stroke: 30](#_Toc107441623)

[S5 Table: Results of the inconsistency analyses of the network: 33](#_Toc107441624)

[S6 Table: Results of the patient population stratified analyses: 34](#_Toc107441625)

[S7 Table: Results of the treatment protocol stratified subgroup analyses: 36](#_Toc107441626)

[Citations of the included trials: 39](#_Toc107441627)

# S1 Figure: Visual estimation of publication bias with funnel plot for stroke events:


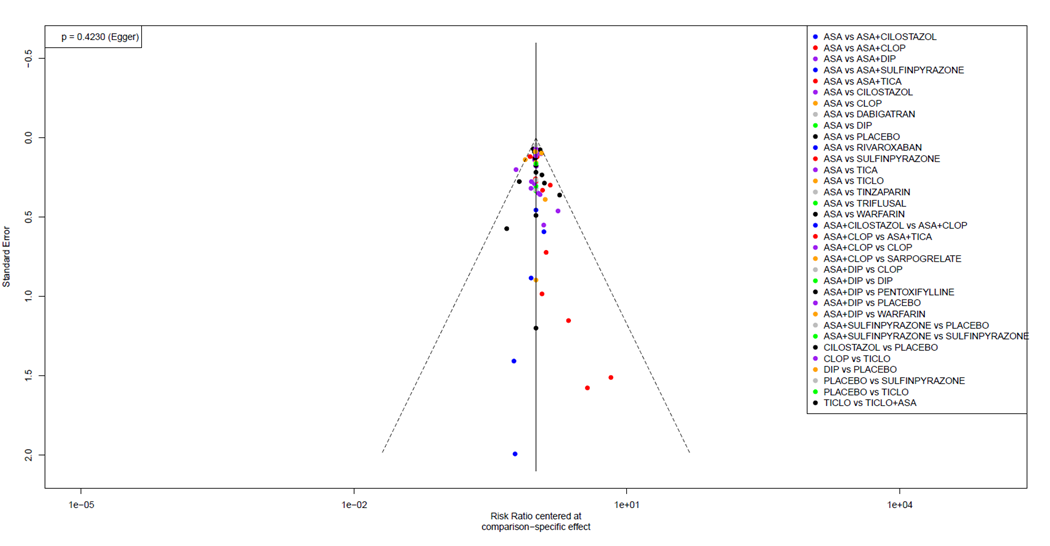
 **S1 Figure legend:** Visual estimation of the funnel plot shows no major asymmetry for publication bias. Egger’s regression test supports no publication bias (p = 0.4230). ***Abbreviations:*** *ASA: aspirin; DIP: dipyridamole; CLOP: clopidogrel; TICA: ticagrelor; TICLO: ticlopidine.*

# S2/A Figure: Bias assessment graph:


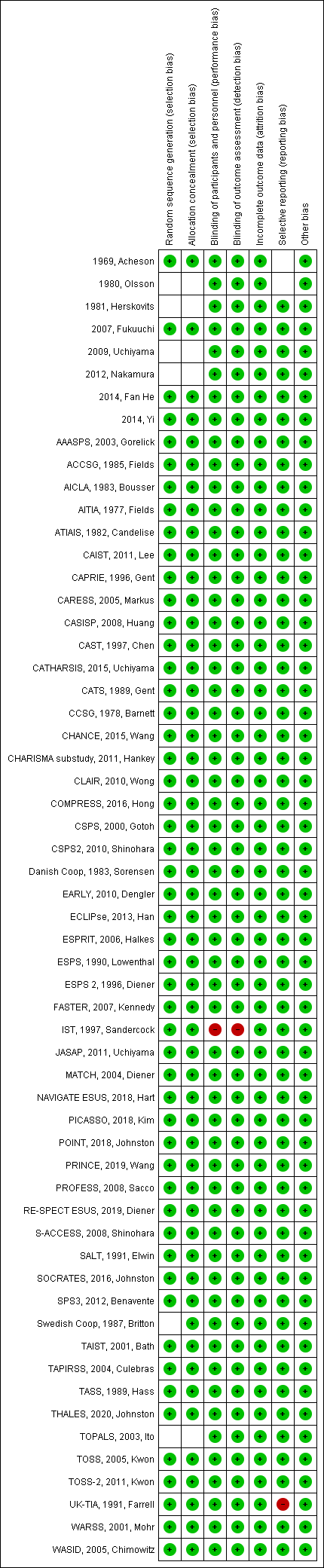


**S2/A Figure legend:** The methodological quality of the included randomized control trials was assessed with the Cochrane Risk Bias tool.

# S2/B Figure: Bias assessment summary report:

*
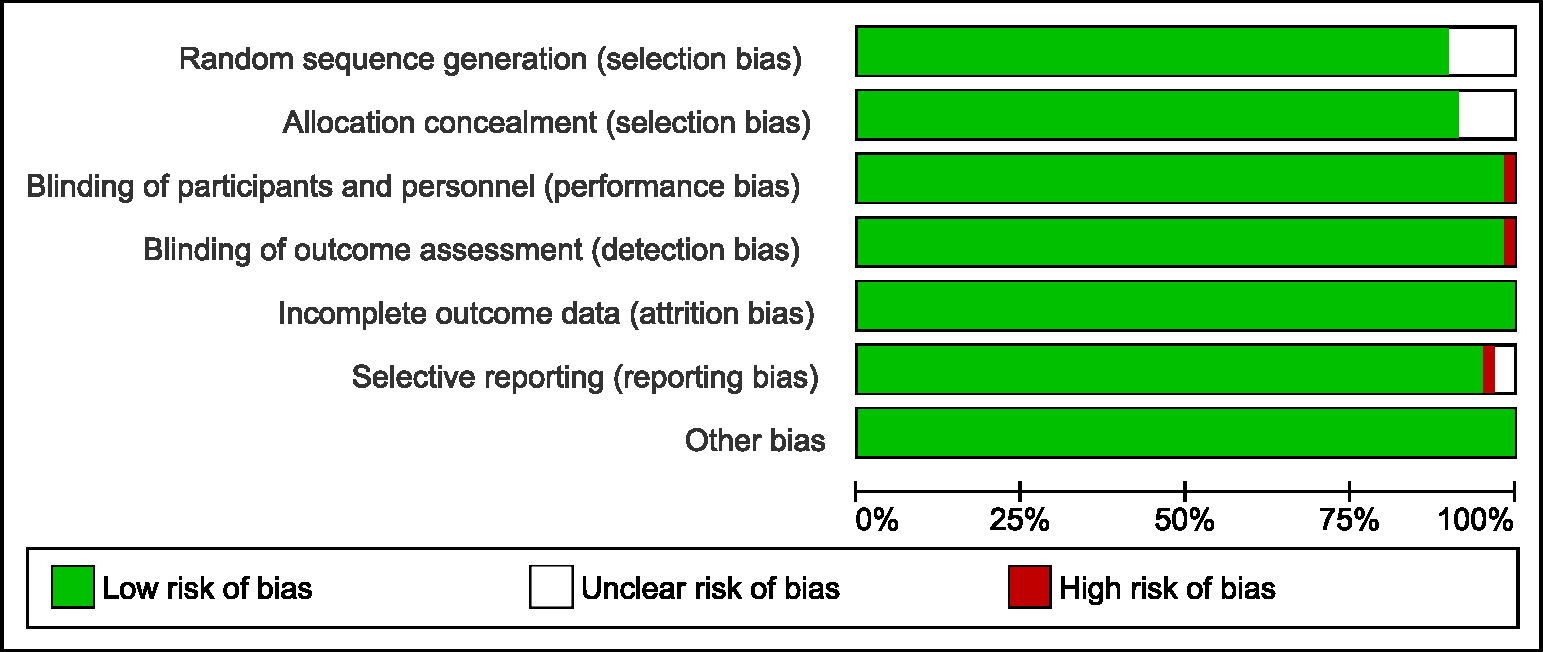
*

**S2/B Figure legend:** The methodological quality of the included randomized control trials was assessed with the Cochrane Risk Bias tool.

# S3 Figure: Supplement heat plot of each intervention:


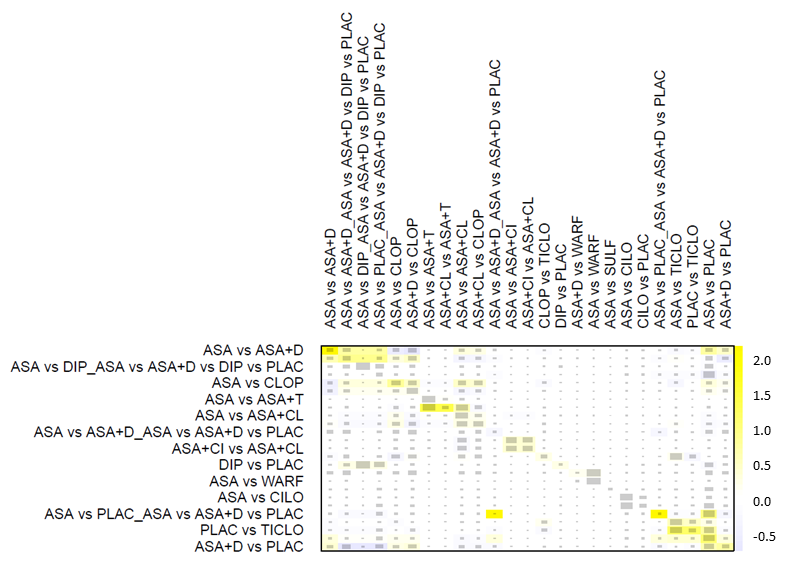


**Abbreviations:** ASA: aspirin; CILO: cilostazol; CLOP, CL: clopidogrel; DIP, D: dipyridamole; PLAC: placebo; SULF: sulfinpyrazone; TICA, T: ticagrelor; TICLO: ticlopidine; WARF: warfarin

# S4 A/B Figures: Analysis of disability outcome:


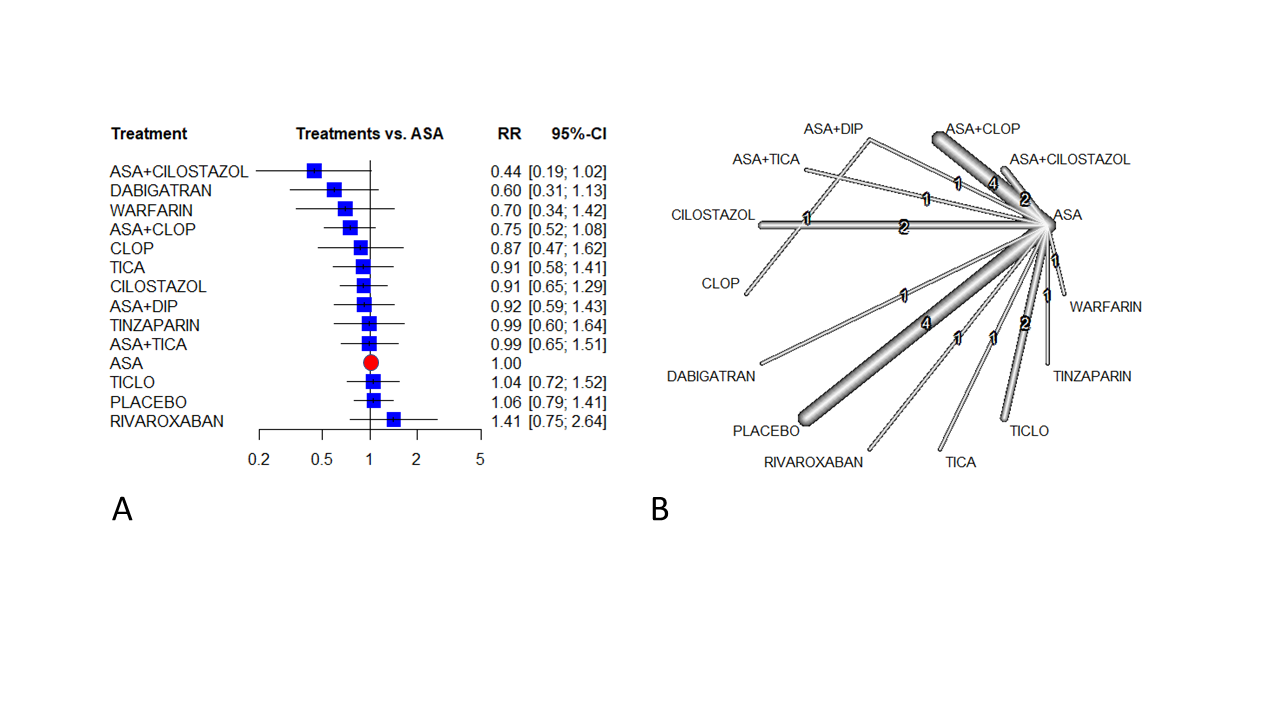


**S4/A figure legend:** Forest plot presents the findings of the random-effects network meta-analysis as contrasting aspirin monotherapy set as reference.

**S4/B figure legend:** Network graph represents the overall composition of evaluations of the disability outcome in our network. The numbers and the width of the edges related to the number of studies within a particular comparison were tested.

**Abbreviations:** ASA: aspirin; CI: confidence interval; CLOP: clopidogrel; DIP: dipyridamole; TICA: ticagrelor; TICLO: ticlopidine; RR: risk ratio

# S1 Table: Characteristics of included trials in our meta-analysis:

| **Trial** | **Author** | **Year** | **Country** | **Patient cohort** | **Treatment** | **No. of Patients** | **Follow up** | **Primary outcome** | | **Inclusion criteria** | **Exclusion criteria** |
| --- | --- | --- | --- | --- | --- | --- | --- | --- | --- | --- | --- |
| **THALES** | Johnston | 2020 | Multiple | TIA/AIS | ticagrelor (180mg) + aspirin (75-100mg) vs. aspirin (75-100mg) | 5523/5493 | 30 days | stroke, death | | - **mild-to-moderate ischemic stroke** - TIA - within 24 hours | - atrial fibrillation - ventricular aneurysm - **cardioembolic cause** - carotid endarterectomy - thrombolysis, thrombectomy - bleeding diathesis - coagulation disorder - history of intracerebral hemorrhage - gastrointestinal bleeding within 6 months - major surgery within 30 days |
| **SOCRATES** | Johnston | 2016 | Multiple | TIA/AIS | ticagrelor (180mg) vs. aspirin (100mg) | 6589/6610 | 90 days | | MACCE | - **non-severe** **ischemic stroke** - TIA - within 24 hours | - atrial fibrillation - ventricular aneurysm - **cardioembolic cause** - thrombolysis, thrombectomy - bleeding diathesis - coagulation disorder - history of intracerebral hemorrhage - gastrointestinal bleeding within 6 months - major surgery within 30 days |
| **PRINCE** | Wang | 2019 | China | TIA/AIS | ticagrelor (180mg) + aspirin (100mg) vs. clopidogrel (75mg) + aspirin (100mg) | 336/339 | 90 days | | HPR  sec.: stroke | - **minor ischemic stroke** - TIA - within 24 hours | - intracranial hemorrhage - acute coronary syndrome - other pathology that could account for the neurological symptoms |
| **SPS3** | Benavente | 2012 | Multiple | TIA/AIS | aspirin (325mg) vs. aspirin (325mg) + clopidogrel (75mg) | 1503/1517 | mean of 3.4 years | | stroke | - symptomatic lacunar stroke - within 180 days | - remote cortical infarct - large subcortical infarct - history of intracerebral, intracranial hemorrhage - disabling stroke - surgically amenable ipsilateral carotid artery disease - **cardioembolic cause** |
| **CAPRIE** | Gent | 1996 | Multiple | AIS | clopidogrel (75mg) vs. aspirin (325mg) | 3233/3198 | mean of 1.91 years | | MACCE | - ischemic stroke - within 1 week and 6 months | - carotid endarterectomy - intracerebral, intracranial hemorrhage - severe cerebral deficit - severe co-morbidity - scheduled for major surgery - severe renal or hepatic insufficiency - hemostatic disorder or systemic bleeding - thrombocytopenia or neutropenia - history of drug-induced hematologic or hepatic abnormalities - abnormal WBC, differential, or platelet count - **anticipated requirement for long-term anticoagulants** |
| **CAST** | Chen | 1997 | China | AIS | aspirin (160mg) vs. placebo | 10335/10320 | 4 weeks | | death | - ischemic stroke - within 48 hours | - gastric bleeding - major life-threatening disease - severe pre-existing disability |
| **ESPS 2** | Diener | 1996 | Multiple | TIA/AIS | aspirin (50mg) vs. dipyridamole (400mg) vs. aspirin (50mg) + dipyridamole (400mg) vs. placebo | 1649/1654/1650/1649 | 2 years | | stroke, death | - TIA - ischemic stroke - within 3 months | - gastrointestinal bleeding - bleeding disturbances - **any condition requiring continued use of ASA or anticoagulants** - life-threatening condition |
| **ESPS** | Lowenthal | 1990 | Multiple | TIA/RIND/AIS | aspirin (990mg) + dipyridamole (225mg) vs. placebo | 1250/1250 | 2 years | | stroke, death | - TIA - RIND - ischemic stroke - within 3 months | - life-threatening associated diseases such as uncontrolled hypertension or diabetes - bleeding diathesis - peptic ulcer |
| **AICLA** | Bousser | 1983 | France | TIA/AIS | placebo vs. aspirin (1000mg) vs aspirin (1000mg) + dipyridamole (225mg) | 204/198/202 | 3 years | | stroke | - TIA - ischemic stroke - within 1 year | - **atrial fibrillation** - **cardiac valvular disease** - polycythemia - thrombocythemia - estrogen treatment - hemodynamic factors - peptic ulcer - ICA or vertebral artery stenosis |
| **MATCH** | Diener | 2004 | Multiple | TIA/AIS | aspirin (75mg) + clopidogrel (75mg) vs. clopidogrel (75mg) | 3797/3802 | 18 months | | MACCE | - ischemic stroke - TIA - within 3 months | - severe comorbid conditions - hepatic insufficiency - peptic ulceration - history of systemic bleeding - bleeding diathesis - coagulopathy - scheduled for major surgery or vascular surgery |
| **PROFESS** | Sacco | 2008 | Multiple | AIS | aspirin (50mg) + dipyridamole (400mg) vs. clopidogrel (75mg) | 10181/10151 | mean of 2.5 years | | stroke | - ischemic stroke - within 90 days | - hemorrhagic stroke - brain tumor - stroke induced by a surgical or cardiovascular procedure such as carotid endarterectomy, angiogram, or cardiac surgery - active peptic ulcer - history of a hemostatic disorder or systemic bleeding |
| **WASID** | Chimowitz | 2005 | North America | TIA/AIS | aspirin (325-1300mg) vs warfarin | 280/289 | mean of 1.8 years | | stroke, brain hemorrhage, CV death | - TIA - **Non-disabling stroke** - within 90 days | - 50 to 99 percent stenosis of the extracranial carotid artery - non-atherosclerotic stenosis of an intracranial artery - **cardiac source of embolism (e.g., atrial fibrillation)** |
| **ESPRIT** | Halkes | 2006 | Multiple | TIA/AIS | aspirin (30-325 mg) + dipyridamole (400mg) vs. aspirin (30-325mg) | 1363/1376 | mean of 3.5 years | | MACCE, major bleeding | - TIA - **minor ischemic stroke** - within 6 months | - **cardiac source of embolism** - recent myocardial infarction - carotid stenosis for which carotid endarterectomy or endovascular treatment was planned - blood coagulation disorder - limited life expectancy |
| **CATS** | Gent | 1989 | North America | AIS | ticlopidine (500mg) vs placebo | 525/528 | mean of 24 months | | MACCE | - ischemic stroke - within 1 week and 4 months | - **cardioembolic cause** - remain bedridden - severe comorbidity - required long-term treatment with anticoagulants or antiplatelet drugs |
| **AAASPS** | Gorelick | 2003 | USA | AIS | ticlopidine (500mg) vs. aspirin (650mg) | 902/907 | average of 1.54 years | | MACCE | - ischemic stroke - within 7 days and 90 days | - TIA - subarachnoid hemorrhage - **cardioembolic source** - iatrogenic stroke - postoperative stroke - carotid endarterectomy - hematologic abnormality - bleeding diathesis - active bleeding - severe comorbid condition - dementia or other neurodegenerative disease - thrombocytopenia, neutropenia - liver or renal failure |
| **CSPS** | Gotoh | 2000 | Japan | AIS | cilostazol (200mg) vs. placebo | 526/526 | mean of 3.2 years | | stroke | - ischemic stroke - within 1 and 6 months | - intracranial hemorrhage - **cardioembolic source** - hemostatic disorders - systemic bleeding - malignant tumor - liver cirrhosis - renal failure - heart failure - bedridden |
| **TASS** | Hass | 1989 | North America | TIA/AIS | ticlopidine (500mg) vs. aspirin (1300mg) | 1529/1540 | 1192±460 days | | death, stroke | - TIA - **minor ischemic stroke** - within 3 months | - **cardiogenic source** - hematologic disorder - history of peptic ulcer - gastrointestinal bleeding - life-threatening disease - **need for continued use of aspirin or anticoagulants** |
| **AITIA** | Fields | 1977 | USA | TIA | aspirin (1300mg) vs. placebo | 88/90 | 6 months | | TIA, stroke, death | - TIA - within 3 months | - bleeding or clotting disorder - history of peptic ulcer - **need for continued use of platelet inhibitors or anticoagulants** |
|  | Herskovits | 1981 | Argentina | TIA | aspirin (1050mg) + dipyridamole (150mg) vs pentoxifylline (1200mg) | 36/30 | 1 year | | stroke, TIA, death | - TIA - within 1 month | - gastric symptoms |
| **COMPRESS** | Hong | 2016 | Korea | AIS | aspirin (100mg) + clopidogrel (75mg) vs. aspirin (100mg) | 174/175 | 30 days | | new ischemic lesion on MRI, sec.: MACCE | - ischemic stroke - within 48 hours | - **cardioembolic stroke** - small-vessel occlusion - history of nontraumatic intracranial bleeding - brain tumor - planned conventional angiography, vascular intervention, or surgery, before the end of the study - bleeding diathesis - coagulopathy |
| **CASISP** | Huang | 2008 | China | AIS | aspirin (100mg) vs. cilostazol (200mg) | 359/360 | 1.5 years | | stroke | - ischemic stroke - within 1 and 6 months | - history of intracranial or subarachnoid hemorrhage - **cardiogenic embolism** - severe disability - uncontrolled severe comorbidities |
| **IST** | Sandercock | 1997 | Multiple | AIS | aspirin (300mg) vs. placebo | 9719/9714 | 14 days | | death | - ischemic stroke - within 48 hours | - intracranial hemorrhage - gastrointestinal bleeding - **already on long-term anticoagulants** |
| **POINT** | Johnston | 2018 | Multiple | TIA/AIS | clopidogrel (75mg) + aspirin (50-325mg) vs. aspirin (50-325mg) | 2432/2449 | 90 days | | MACCE | - TIA - **minor ischemic stroke** - within 12 hours | - thrombolysis - endovascular therapy or endarterectomy - **atrial fibrillation** - **cardiovascular disease in whom anticoagulation would be indicated** |
| **FASTER** | Kennedy | 2007 | North America | TIA/AIS | clopidogrel (75mg) + aspirin (81mg) vs. aspirin (81mg) | 198/194 | 90 days | | stroke | - TIA - **minor ischemic stroke** - within 24 hours | - intracranial hemorrhage - thrombolysis, thrombectomy - **cardiac source** - acute coronary syndrome - secondary to a procedure - limited life expectancy |
| **PICASSO** | Kim | 2018 | Asia | TIA/AIS | cilostazol (200mg) vs. aspirin (100mg) | 755/757 | median of 1.9 years | | MACCE | - ischemic stroke or TIA with a history of imaging findings of intracerebral hemorrhage or two or more microbleeds - within 180 days | - cerebral hemorrhage within 6 months before study entry - **cardioembolic source** - severe cardiomyopathy - congestive heart failure - myocardial infarction within 4 weeks |
| **CAIST** | Lee | 2011 | South Korea | AIS | cilostazol (200mg) vs. aspirin (300mg) | 231/227 | 90 days | | mRS  sec.: MACCE | - ischemic stroke - within 48 hours | - **cardioembolic source** - congestive heart failure - severe uncontrolled hypertension or hypotension - thrombolysis - intracranial mass - anemia - thrombocytopenia - abnormal liver and renal function |
| **EARLY** | Dengler | 2010 | Germany | TIA/AIS | aspirin (25mg) + dipyridamole (400mg) vs aspirin (100mg) | 283/260 | 7 days | | mRS  sec.: MACCE | - ischemic stroke - TIA - within 24 hours | - intracranial hemorrhage - thrombolysis - active gastric or duodenal ulcers - bleeding disorders - **indication for anticoagulation** |
| **TOSS** | Kwon | 2005 | South Korea | AIS | cilostazol (200mg) + aspirin (100mg) vs. aspirin (100mg) | 67/68 | 6 months | | progression of symptomatic stenosis on MRI  sec.: MACCE | - ischemic stroke - within 2 weeks | - **cardioembolic source** - stenosis of extracranial arteries - bleeding diatheses - recent major bleeding - chronic devastating illness - thrombocytopenia - anemia |
| **WARSS** | Mohr | 2001 | USA | AIS | warfarin vs. aspirin (325mg) | 1103/1103 | 2 years±1 month | | ischemic stroke, death | - ischemic stroke - within 30 days | - due to a procedure - carotid stenosis for which surgery was planned - **cardioembolic source** |
| **SALT** | Elwin | 1991 | Sweden | TIA/AIS | aspirin (75mg) vs placebo | 676/684 | median of 32 months | | stroke, death | - **minor ischemic stroke** - TIA - within 3 months | - **cardiac source of emboli** - previous or planned carotid surgery - other causes of the symptoms established (arteritis, dissection, migraine, hematological or hyperviscosity disorders, or orthostatically induced symptoms only) - other severe disorders - **need for long-term treatment with anticoagulant or antiplatelet drugs** |
|  | Nakamura | 2012 | Japan | AIS | aspirin (100mg) vs. aspirin (100mg) + cilostazol (200mg) | 38/38 | 6 months | | stroke | - **minor ischemic stroke** - within 48 hours | - **cardioembolic source** - acute intracranial hemorrhage - **indication for anticoagulation** - thrombolysis |
| **CSPS2** | Shinohara | 2010 | Japan | AIS | cilostazol (200mg) vs. aspirin (81mg) | 1337/1335 | mean of 29 months | | stroke | - ischemic stroke - within 26 weeks | - **cardioembolic source** - increased risk of hemorrhage - congestive heart failure - peptic ulcer - blood, hepatic, or renal disorders - undergone or scheduled to undergo percutaneous transluminal angioplasty or revascularization |
| **CHARISMA substudy** | Hankey | 2011 | Multiple | TIA/AIS | clopidogrel (75mg) + aspirin (75-162mg) vs. aspirin (75-162mg) | 2157/2163 | median of 25 months | | stroke | - ischemic stroke - TIA - within 5 years | - taking oral antithrombotic medications or nonsteroidal anti-inflammatory drugs on a long-term basis |
| **CARESS** | Markus | 2005 | Multiple | TIA/AIS | clopidogrel (75mg) + aspirin (75mg) vs. aspirin (75mg) | 51/56 | 7 days | | MES positive  sec.: cerebrovascular events | - 50% carotid stenosis, and had experienced ipsilateral carotid territory TIA or stroke - within 3 months | - hemorrhagic transformation - atrial fibrillation - **cardiac source of embolism** - thrombolysis - anticoagulation within the last 3 days - thrombocytopenia - bleeding diathesis - coagulopathy - neutropenia |
| **Danish Coop** | Sorensen | 1983 | Denmark | TIA/RIND/AIS | aspirin (1000mg) vs. placebo | 101/102 | median of 25 months | | stroke, death | - TIA - RIND - within 1 month | - residual symptoms from a previous stroke - bad physical condition due to other illness - peptic ulcer - referred to carotid surgery |
| **CATHARSIS** | Uchiyama | 2015 | Japan | AIS | cilostazol (200mg) + aspirin (100mg) vs. aspirin (100mg) | 83/82 | mean of 762 days | | progression of stenosis on MRA  sec.: MACCE | - ischemic stroke patients with >50% stenosis in the responsible intracranial artery - within 2 weeks and 6 months | - **cardiac sources of embolism** - history of symptomatic intracranial hemorrhage - other hemorrhagic diseases - hemophilia or coagulation abnormalities - liver and renal dysfunction - scheduled to undergo percutaneous transluminal angioplasty or bypass surgery |
| **UK-TIA** | Farrell | 1991 | UK | TIA/AIS | aspirin (300mg-1200mg) vs placebo | 1621/814 | mean of 4 years | | MACCE | - TIA, - **minor ischemic** **stroke** - within 3 months - patients were included with cardiac sources of embolism who were not anticoagulated | - history of previous disabling major stroke - due to something other than arterial thromboembolism - renal failure - peptic ulcer - abnormal bleeding - myocardial infarction within 3 months |
| **CHANCE** | Wang | 2015 | China | TIA/AIS | clopidogrel (75mg) + aspirin (75mg) vs. aspirin (75mg) | 2584/2586 | 1 year | | stroke | - **minor ischemic stroke** - TIA - within 24 hours | - hemorrhage - vascular malformation - tumor, abscess - other major nonischemic brain diseases - **cardiac sources of embolism** - clear indication for anticoagulation - thrombolysis |
| **CLAIR** | Wong | 2010 | Asia | TIA/AIS | clopidogrel (75mg) + aspirin (75mg-160mg) vs. aspirin (75mg-160mg) | 46/52 | 7 days | | MES on day 2  sec.: stroke, death, bleeding | - TIA - **minor ischemic stroke** - with symptomatic large artery stenosis in the cerebral or carotid arteries and in whom MES were present - within 7 days | - intracerebral hemorrhage - brain tumor - extracranial or intracranial internal carotid artery or middle cerebral artery stenosis - history of intracerebral hemorrhage - anticoagulation therapy before the onset of stroke - definite indication for anticoagulation - terminal carcinoma - renal failure - cirrhosis - severe dementia - psychosis - **atrial fibrillation** - **rheumatic heart disease or metallic heart valve** - thrombocytopenia |
|  | Fan He | 2014 | China | TIA/AIS | clopidogrel (75mg) + aspirin (100mg) vs. aspirin (300mg) | 321/326 | 14 days | | stroke | - TIA - **minor ischemic stroke** - within 72 hours | - **cardioembolic source** - thrombocytopenia - anticoagulation therapy before stroke onset - definite indication for anticoagulation - current peptic ulceration - history of systemic bleeding - coagulopathy - major surgery or trauma in the previous 3 months - terminal malignancy - serious renal or liver disease - thrombolysis |
|  | Yi | 2014 | China | AIS | clopidogrel (75mg) + aspirin (200mg) vs. aspirin (200mg) | 284/286 | 30 days | | MACCE | - ischemic stroke - within 48 hours | - history of carotid endarterectomy or carotid stent therapy - clinically relevant arrhythmia - atrial fibrillation - liver, renal failure - severe cardiovascular disease - malignancies - thrombocytopenia - anemia - major surgical procedure within 1 week - history of myeloproliferative disorders - heparin-induced thrombocytopenia |
|  | Acheson | 1969 | UK | TIA/AIS | dipyridamole (400mg->800mg) vs. placebo | 85/84 | 25 months | | stroke, death | - ischemic stroke - TIA - within 5 years | - NA |
| **ACCSG** | Fields | 1985 | North America | TIA | dipyridamole (300mg) + aspirin (1300mg) vs. aspirin (1300mg) | 448/442 | median of 25 months | | stroke, death | - TIA - within 3 months | - serious concurrent illnesses - take anticoagulants or other drugs that influenced platelet function - carotid endarterectomy - **cardiac source for embolism** - history of peptic ulcer - bleeding or clotting disorder |
| **CCSG** | Barnett | 1978 | Canada | TIA | aspirin (1300mg) vs. placebo vs. sulfinpyrazone (800mg) vs. aspirin (1300mg) + sulfinpyrazone (800mg | 144/139/156/146 | mean of 26 months | | TIA, stroke, death | - TIA - within 3 months | - coexisting morbid condition explaining their symptoms - likely to die from other illness within 12 months |
|  | Fukuuchi | 2007 | Japan | AIS | clopidogrel (75mg) vs ticlopidine (200mg) | 573/578 | 52 weeks | | safety: hematologic changes, hepatic dysfunction, nontraumatic hemorrhage, serious adverse drug reactions  sec.: MACCE | - ischemic stroke - from 8 days with no maximum time limit | - **cardioembolic source** - bleeding disorders - risk of bleeding - history of intracranial hemorrhage - current diabetic retinopathy - severe renal or heart disease - uncontrolled hypertension |
| **ECLIPse** | Han | 2013 | Korea | AIS | cilostazol (200mg) + aspirin (100mg) vs. aspirin (100mg) | 100/103 | 90 days | | middle cerebral artery and basilar artery pulsatility index  sec.: stroke | - ischemic stroke - within 7 days | - **cardiac sources of embolism** - bleeding diathesis - recent history of major bleeding - chronic liver or chronic renal disease - anemia - thrombocytopenia - any non-atherosclerotic vasculopathy, such as arterial dissection, Moya Moya disease, Takayasu's arteritis, radiation-associated angiopathy, or other vasculitis - hyperthyroidism or - chronic obstructive pulmonary disease - current anticoagulation or antiplatelet therapy |
| **TOSS-2** | Kwon | 2011 | East Asia | AIS | cilostazol (200mg) + aspirin (75-150mg) vs. clopidogrel (75mg) + aspirin (75-150mg) | 232/225 | 7 months | | progression of intracranial atherosclerotic stenosis  sec.: MACCE | - ischemic stroke - within 2 weeks | - nonatherosclerotic vasculopathy such as arterial dissection or Moya Moya disease - thrombolytic therapy - **embolic heart disease** - significant stenosis of arteries proximal to the symptomatic stenosis - scheduling for revascularization for the stenosis |
| **Swedish Coop** | Britton | 1987 | Sweden | AIS | aspirin (1500mg) vs placebo | 253/252 | 2 years | | stroke, death | - ischemic stroke - within 1 and 3 weeks | - **atrial fibrillation** - recent myocardial infarction - **other needs for anticoagulant or** **antiplatelet drugs** - peptic ulcer disease - malignancy - other serious diseases |
|  | Uchiyama | 2009 | Japan | AIS | clopidogrel (75mg) vs ticlopidine (200mg) | 939/923 | 52 weeks | | safety: hematologic changes, hepatic dysfunction, and atraumatic serious hemorrhage  sec.: MACCE | - ischemic stroke - from 8 days with no maximum time limit | - **cardiac sources of embolism** - TIA after the recent stroke - serious impairment - bleeding disorders - history of intracranial hemorrhage - severe renal - heart disease - uncontrolled hypertension - hepatic dysfunction - thrombocytopenia - leukopenia |
| **JASAP** | Uchiyama | 2011 | Japan | AIS | dipyridamole (400mg) + aspirin (50mg) vs. aspirin (81mg) | 652/639 | mean of 15.6 months | | stroke | - ischemic stroke - within 1 week and 6 months | - diagnosis of brain disorders with a bleeding risk - **cardiogenic sources of embolism** - acute coronary syndromes < 6 months - history of peptic ulcer < 3 years - having undergone arterial reconstruction after developing ischemic stroke - severe disability - bleeding or bleeding tendencies - serious cardiac, renal or hepatic disorders - malignant tumor or having received cancer treatment in the past 5 years |
| **S-ACCESS** | Shinohara | 2008 | Japan | AIS | sarpogrelate (100mg) vs. aspirin (81mg) | 747/752 | mean of 1.59 years | | stroke | - ischemic stroke - within 6 months | - **cardioembolic stroke** - mRS score of 4 or more - previous or scheduled vascular surgery for cerebral infarction - history of intracranial hemorrhage - systemic bleeding - peptic ulcer - history of bleeding diathesis or coagulopathy - severe complications such as cardiac, renal, hepatic, and blood disorders - treatment for malignancy within the past 5 years |
| **ATIAIS** | Candelise | 1982 | Italy | TIA | aspirin (1000mg) vs. sulfinpyrazone (800mg) | 63/61 | mean of 11.23 months | | MACCE | - TIA - within 3 months | - previous peptic ulcer - marked renal or hepatic insufficiency - other life-limiting diseases - **cardiac or hemodynamic causes** - take drugs with antiplatelet or anticoagulant action |
|  | Olsson | 1980 | Sweden | TIA/RIND | aspirin (1000mg) + dipyridamole (150mg) vs. warfarin | 67/68 | 1-19 months | | MACCE | - TIA - RIND - within 90 days | - history of cardiac disease - severe peripheral arterial insufficiency |
| **TAIST** | Bath | 2001 | Multiple | AIS | tinzaparin (100IU/kg-175IU/kg) vs. aspirin (300mg) | 993/491 | 10 days | | stroke, death, bleeding | - ischemic stroke - within 48 h | - intracranial hemorrhage - coma - mild stroke - stroke complicating trauma or a medical or surgical procedure - stroke or myocardial infarction within the previous 3 months - congenital bleeding disorder - anemia - thrombocytopenia - liver and renal dysfunction - endocarditis - **recent anticoagulant therapy or need for anticoagulation** - thrombolysis - severe concomitant medical conditions |
| **TAPIRSS** | Culebras | 2004 | Multiple | TIA/AIS | triflusal (600mg) vs. aspirin (325mg) | 213/216 | 2 years | | MACCE | - TIA - **minor ischemic stroke** - within 15 days and 6 months | - disabling stroke - brain hemorrhage - stroke of non-atherothrombotic cause - **cardioembolic source of stroke** - previous carotid endarterectomy - cognitive impairment - renal or liver failure - moderate or severe heart failure - HIV infection - alcohol or drug abuse - active peptic ulcer - need for long-term anticoagulant, or antiplatelet agents - malignancy with high bleeding risk |
| **RE-SPECT ESUS** | Diener | 2019 | Multiple | AIS | dabigatran (220-300mg) vs. aspirin (100mg) | 2695/2695 | median of 19 months | | stroke, bleeding | - **minor ischemic stroke** - within 3 months | - **cardioembolic source** - intracerebral hemorrhage - increased risk of bleeding - mRS score of ≥4 at the time of randomization - other specific stroke etiology (e.g., cerebral arteritis or arterial dissection, migraine/vasospasm, drug abuse) - renal failure |
| **NAVIGATE ESUS** | Hart | 2018 | Multiple | AIS | rivaroxaban (15mg) vs aspirin (100mg) | 3609/3604 | median of 11 months | | stroke, bleeding | - **minor ischemic stroke** - between 7 days and 6 months | - extracranial vessel atherosclerosis causing more than 50% luminal stenosis - **cardiac source of embolism** - severely disabling stroke - indication for anticoagulation or antiplatelet therapy - major bleeding within the previous 6 months - previous nontraumatic intracranial hemorrhage |
| **TOPALS** | Ito | 2003 | Japan | TIA/AIS | ticlopidine (200mg) vs. ticlopidine (100mg) + aspirin (81mg) | 138/132 | average of1.59 years | | MACCE | - ischemic stroke within 1 and 6 months - TIA within the previous 3 months | - history of a cerebral hemorrhage - **cardiogenic cerebral infarction** - severe hepatic or renal dysfunction - bleeding tendency |

**Abbreviations:** AIS: acute ischemic event, ASA: aspirin, CV: cardiovascular, HPR: high platelet reactivity, ICA: internal carotid artery, MACCE: major adverse cardiac and cerebrovascular events, MES: microembolic signals, MRI: magnetic resonance imaging, mRS: modified Rankin Scale, NA: not applicable, RIND: reversible ischemic neurological deficit, TIA: transient ischemic attack, WBC: white blood count

**cardiogenic source:** mitral valve stenosis, prosthetic valve, endocarditis, valvular vegetations, myocardial infarction within 6 weeks, ventricular aneurysm intraventricular or intraatrial blood clots, mitral valve prolapse, atrial fibrillation, sick sinus syndrome, idiopathic cardiomyopathy, atrial myxoma or other cardiac tumors

# S2 Table: Clinical characteristics of the included patient populations:

| **Acronym**  **year**  **author** | **Blinding** | **Patient cohort** | **Length of treatment period** | **No.**  **sample size** | **No. (%)**  **female sex** | **Mean age**  **(years)** | **No. (%)**  **prior TIA** | | **No. (%)**  **prior stroke** | **No. (%)**  **hypertension** | **No. (%)**  **PAD** | **No. (%)**  **prior MI** | **No. (%)**  **DM** | **No. (%)**  **dyslipidemia** |
| --- | --- | --- | --- | --- | --- | --- | --- | --- | --- | --- | --- | --- | --- | --- |
| **THALES**  **2020**  **Johnston** | double blind | TIA/AIS | 30 days | 11016 | 4279 (39) | 65 | 515 (5) | | 1815 (17) | 8520 (77) | NA | NA | 3142 (29) | NA |
| **SOCRATES**  **2016**  **Johnston** | double blind | TIA/AIS | 90 days | 13199 | 5483 (42) | 66 | | 856 (6) | 1593 (12) | 9730 (74) | NA | 548 (4) | 3212 (24) | 5028 (38) |
| **PRINCE**  **2019**  **Wang** | open label treatment / blinded endpoints | TIA/AIS | 90 days | 675 | 181 (27) | 61 | | 18 (3) | 121 (18) | 411 (61) | NA | NA | 164 (24) | 41 (6) |
| **SPS3**  **2012**  **Benavente** | double blind | TIA/AIS | mean of 3.4 years | 3020 | 1117 (37) | 63 | | 452 (15) | | 2264 (75) | NA | NA | 1102 (36) | NA |
| **CAPRIE**  **1996**  **Gent** | double blind | AIS | mean of 1.63 year | 6431 | 2346 (36) | 65 | | 1221 (19) | 1157 (18) | 4179 (65) | NA | 772 (12) | 1639 (25) | 2411 (0.37) |
| **CAST**  **1997**  **Chen** | double blind | AIS | 4 weeks | 21106 | 7739 (37) | 63 | | NA | NA | NA | NA | NA | NA | NA |
| **ESPS 2**  **1996**  **Diener** | double blind | TIA/AIS | 2 years | 6602 | 2774 (42) | 67 | | NA | NA | 3997 (60) | 1454 (22) | NA | 1011 (15) | 1509 (23) |
| **ESPS**  **1990**  **Lowenthal** | double blind | TIA/RIND/AIS | 2 years | 2500 | 1050 (42) | 63 | | NA | NA | 916 (36) | NA | NA | NA | NA |
| **AICLA**  **1983**  **Bousser** | double blind | TIA/AIS | 3 years | 604 | 181 (30) | 63 | | NA | NA | 380 (63) | 42 (7) | 42 (7) | 132 (22) | 157 (26) |
| **MATCH**  **2004**  **Diener** | double blind | TIA/AIS | 18 months | 7599 | 2821 (37) | 66 | | 1442 (19) | 1981 (26) | 5945 (78) | 776 (10) | 363 (5) | 5197 (68) | 4280 (56) |
| **PROFESS**  **2008**  **Sacco** | double blind | AIS | mean of 2.5 years | 20332 | 7319 (36) | 66 | | 1769 (8) | 3709 (18) | 15045 (74) | 609 (3) | 1362 (7) | 5743 (28) | 9474 (46) |
| **WASID**  **2005**  **Chimowitz** | double blind | TIA/AIS | mean of 1.8 years | 569 | 219 (38) | 64 | | NA | 138 (24) | 477 (84) | NA | NA | 216 (38) | 391 (69) |
| **ESPRIT**  **2006**  **Halkes** | open label treatment / blinded endpoints | TIA/AIS | mean of 3.5 years | 2739 | 950 (35) | 63 | | NA | 314 (11) | 1631 (59) | 128 (5) | 184 (7) | 512 (19) | 1272 (46) |
| **CATS**  **1989**  **Gent** | double blind | AIS | mean of 18 months | 1053 | 406 (38) | 65 | | 183 (17) | 821 (78) | 710 (67) | 110 (10) | 179 (17) | 331 (31) | NA |
| **AAASPS**  **2003**  **Gorelick** | double blind | AIS | average of 1.54 years | 1809 | 967 (53) | 61 | | NA | NA | 1539 (85) | NA | 170 (9) | 738 (41) | 697 (38) |
| **CSPS**  **2000**  **Gotoh** | double blind | AIS | 663±461 days | 1052 | 684 (65) | 65 | | NA | NA | 636 (60) | NA | NA | 258 (25) | 252 (24) |
| **TASS**  **1989**  **Hass** | double blind | TIA/AIS | 818±592 days | 3069 | 1082 (35) | 63 | | NA | 295 (10) | 1204 (39) | 447 (14) | 514 (17) | 597 (19) | 1122 (36) |
| **AITIA**  **1977**  **Fields** | double blind | TIA | 6 months | 178 | 60 (34) | NA | | NA | 16 (9) | 84 (47) | 16 (9) | 33 (18) | 25 (14) | NA |
| **1981**  **Herskovits** | open label | TIA | 1 year | 66 | 16 (24) | 60 | | NA | NA | 39 (59) | NA | NA | 11 (16) | 48 (72) |
| **COMPRESS**  **2016**  **Hong** | double blind | AIS | 30 days | 349 | 127 (36) | 68 | | NA | 36 (10) | 231 (66) | NA | NA | 113 (32) | 109 (31) |
| **CASISP**  **2008**  **Huang** | double blind | AIS | 12-18 months | 719 | 225 (31) | 60 | | NA | NA | 569 (79) | NA | NA | 131 (18) | 211 (29) |
| **IST**  **1997**  **Sandercock** | open label | AIS | 14 days | 19433 | NA | NA | | NA | NA | NA | NA | NA | NA | NA |
| **POINT**  **2018**  **Johnston** | double blind | TIA/AIS | 90 days | 4881 | 2195 (45) | 65 | | NA | NA | 3373 (69) | NA | NA | 1340 (27) | NA |
| **FASTER**  **2007**  **Kennedy** | double blind | TIA/AIS | 90 days | 392 | 185 (47) | 68 | | 63 (16) | 29 (7) | 198 (50) | 8 (2) | 19 (4) | 42 (10) | 28 (7) |
| **PICASSO**  **2018**  **Kim** | double blind | AIS | median of 1.9 years | 1512 | 575 (38) | 65 | | NA | NA | 1347 (89) | NA | NA | 485 (32) | 661 (44) |
| **CAIST**  **2011**  **Lee** | double blind | AIS | 90 days | 458 | 177 (38) | 63 | | NA | NA | 297 (65) | NA | NA | 159 (35) | 189 (41) |
| **EARLY**  **2010**  **Dengler** | open label treatment / blinded endpoints | TIA/AIS | 7 days | 543 | 204 (37) | 68 | | NA | 77 (14) | 402 (74) | NA | NA | 129 (24) | 182 (33) |
| **TOSS**  **2005**  **Kwon** | double blind | AIS | 6 months | 135 | 53 (39) | 62 | | NA | NA | 78 (58) | NA | NA | 54 (40) | 17 (13) |
| **WARSS**  **2001**  **Mohr** | double blind | AIS | 2 years±1 months | 2206 | 897 (41) | 63 | | 629 (29) | | 1499 (68) | NA | NA | 705 (32) | NA |
| **SALT**  **1991**  **Elwin** | double blind | TIA/AIS | mean of 30 and 27 months | 1360 | 470 (34) | 67 | | 128 (9) | 124 (9) | 632 (46) | 111 (8) | 149 (11) | 175 (13) | NA |
| **2012**  **Nakamura** | open label | AIS | 6 months | 76 | 20 (26) | 67 | | NA | NA | 62 (81) | NA | NA | 26 (34) | 23 (30) |
| **CSPS2**  **2010**  **Shinohara** | double blind | AIS | 1-5 years | 2672 | 756 (28) | 63 | | NA | NA | 1967 (74) | NA | NA | 775 (29) | 1159 (43) |
| **CHARISMA substudy**  **2011**  **Hankey** | double blind | TIA/AIS | median of 25 months | 4320 | 1576 (36) | 64 | | 1410 (32) | 3332 (77) | 3295 (76) | 259 (6) | NA | 1253 (29) | NA |
| **CARESS**  **2005**  **Markus** | double blind | TIA/AIS | 7 days | 107 | 33 (30) | 64 | | NA | 7 (6) | 69 (64) | 17 (16) | 16 (15) | 34 (32) | 60 (56) |
| **Danish Coop**  **1983**  **Sorensen** | double blind | TIA/RIND/AIS | median of 25 months | 203 | 55 (27) | 59 | | NA | 11 (5) | 55 (27) | 30 (15) | 18 (8) | NA | 59 (29) |
| **CATHARSIS**  **2015**  **Uchiyama** | open label | AIS | 2 years | 163 | 56 (34) | 68 | | NA | NA | 124 (76) | NA | NA | 60 (37) | 89 (54) |
| **UK-TIA**  **1991**  **Farrell** | double blind | TIA/AIS | mean of 4 years | 2435 | 656 (27) | 60 | | NA | 81 (3) | 955 (39) | 298 (12) | 242 (10) | 106 (4) | 856 (35) |
| **CHANCE**  **2015**  **Wang** | double blind | TIA/AIS | 21 days | 5170 | 1750 (34) | 62 | | 174 (3) | 1033 (20) | 3399 (66) | NA | 96 (2) | 1093 (21) | 573 (11) |
| **CLAIR**  **2010**  **Wong** | open label treatment / blinded endpoint | TIA/AIS | 7 days | 98 | 22 (22) | 57 | | NA | NA | 62 (63) | 6 (6) | 6 (6) | 37 (38) | 39 (40) |
| **2014**  **Fan He** | open label | TIA/AIS | 14 days | 647 | 279 (43) | 62 | | 221 (34) | | 437 (67) | NA | NA | 266 (41) | NA |
| **2014**  **Yi** | open label | AIS | 30 days | 570 | 257 (45) | 70 | | NA | NA | 414 (73) | NA | 7 (1) | 215 (38) | NA |
| **1969**  **Acheson** | double blind | TIA/AIS | 25 months | 169 | 52 (31) | 58 | | NA | NA | 97 (57) | NA | NA | NA | NA |
| **ACCSG**  **1985**  **Fields** | double blind | TIA | median of 25 months | 890 | 294 (33) | 63 | | NA | 116 (13) | 423 (48) | 69 (8) | 134 (15) | 132 (15) | NA |
| **CCSG**  **1978**  **Barnett** | double blind | TIA | mean of 26 months | 585 | 179 (31) | NA | | NA | NA | NA | NA | NA | NA | NA |
| **2007**  **Fukuuchi** | double blind | AIS | 52 weeks | 1151 | 311 (27) | 64 | | NA | NA | 780 (68) | NA | NA | 434 (38) | 222 (19) |
| **ECLIPse**  **2013**  **Han** | double blind | AIS | 90 days | 203 | 51 (25) | 65 | | NA | NA | 116 (57) | NA | NA | 58 (28) | 30 (15) |
| **TOSS-2**  **2011**  **Kwon** | double blind | AIS | 7 months | 457 | 223 (49) | 65 | | NA | NA | 331 (72) | NA | NA | 194 (42) | 217 (47) |
| **Swedish Coop**  **1987**  **Britton** | double blind | AIS | 2 years | 505 | 192 (38) | 68 | | 40 (8) | 55 (11) | 232 (46) | 45 (9) | 50 (10) | 86 (17) | 10 (2) |
| **2009**  **Uchiyama** | double blind | AIS | 26 -52 weeks | 1862 | 529 (28) | 64 | | NA | NA | 1304 (70) | NA | NA | 410 (22) | 661 (35) |
| **JASAP**  **2011**  **Uchiyama** | double blind | AIS | 447-471 days | 1294 | 369 (28) | 66 | | NA | NA | 1143 (88) | NA | NA | 523 (40) | 856 (66) |
| **S-ACCESS**  **2008**  **Shinohara** | double blind | AIS | mean of 580 days | 1499 | 423 (28) | 65 | | NA | 200 (13) | 1037 (69) | NA | NA | 419 (28) | 593 (39) |
| **ATIAIS**  **1982**  **Candelise** | double blind | TIA | mean of 11.23 months | 124 | 38 (31) | 54 | | NA | NA | 58 (47) | 11 (9) | NA | 9 (7) | 56 (45) |
| **1980**  **Olsson** | open label | TIA/RIND/AIS | 1-19 months | 135 | 42 (31) | 66 | | NA | 25 (18) | 65 (48) | NA | 11 (8) | 16 (12) | 8 (6) |
| **TAIST**  **2001**  **Bath** | double blind | AIS | 10 days | 1486 | 678 (46) | 74 | | 242 (16) | 194 (13) | 728 (49) | 94 (6) | 232 (16) | 250 (17) | 226 (15) |
| **TAPIRSS**  **2004**  **Culebras** | double blind | TIA/AIS | mean of 586 days | 429 | 136 (32) | 65 | | 67 (16) | 55 (13) | 303 (71) | 15 (3) | 24 (5) | 78 (18) | 160 (37) |
| **RE-SPECT ESUS**  **2019**  **Diener** | double blind | AIS | median of 19 months | 5390 | 1987 (37) | 64 | | 975 (18) | | 3981 (74) | NA | 340 (6) | 1224 (23) | 3043 (56) |
| **NAVIGATE ESUS**  **2018**  **Hart** | double blind | AIS | median of 11 months | 7213 | 2777 (38) | 67 | | 1263 (17) | | 5585 (77) | NA | NA | 1806 (25) | NA |
| **TOPALS**  **2003**  **Ito** | open label | TIA/AIS | average of 1.59 years | 270 | 95 (35) | 67 | | NA | NA | 128 (47) | NA | NA | 61 (23) | 27 (10) |

***Abbreviations:*** *AIS: acute ischemic event, DM: diabetes mellitus, MI: myocardial infarction, NA: not applicable, PAD: peripheral artery disease, RIND: reversible ischemic neurological deficit, TIA: transient ischemic attack*

# S3 A/B Tables: Summary tables of the GRADEpro evaluation of the included cilostazol studies:

**S3/A Table: summarizes the certainty of the cilostazol vs. aspirin studies:**

| **Certainty assessment** | | | | | | | **№ of patients** | | **Effect** | | **Certainty** | **Importance** |
| --- | --- | --- | --- | --- | --- | --- | --- | --- | --- | --- | --- | --- |
| **№ of studies** | **Study design** | **Risk of bias** | **Inconsistency** | **Indirectness** | **Imprecision** | **Other considerations** | **cilostazol** | **aspirin** | **Relative (95% CI)** | **Absolute (95% CI)** |  |  |
| **Stroke** | | | | | | | | | | | | |
| 4 | randomized trials | not serious | serious^a^ | not serious | not serious | none | 147/2683 (5.5%) | 221/2678 (8.3%) | **RR 1.50** (1.23 to 1.84) | **41 more per 1,000** (from 19 more to 69 more) | **⨁⨁⨁◯ Moderate** |  |
| **Mortality** | | | | | | | | | | | | |
| 2 | randomized trials | not serious | not serious | not serious | serious^b^ | none | 41/2092 (2.0%) | 44/2092 (2.1%) | not estimable |  | **⨁⨁⨁◯ Moderate** |  |
| **Bleeding** | | | | | | | | | | | | |
| 4 | randomized trials | not serious | serious^c^ | not serious | not serious | none | 229/2691 (8.5%) | 351/2682 (13.1%) | not estimable |  | **⨁⨁⨁◯ Moderate** |  |

**S3/A Table legend:** In the studies comparing cilostazol vs. aspirin the stroke, mortality, and bleeding outcomes, according to the GRADEpro evaluation, reflected in moderate certainty.

a. In PICASSO and CSPS2 trials, there was a significant difference between aspirin and cilostazol in the prevention of recurrent stroke in favor of cilostazol, in CAIST and CASISP studies, there was no significant difference. However, confidence intervals are nearly overlapping, and the risk ratio reductions are comparable.

b. Only in the CSPS and PICASSO studies was mortality examined as an endpoint, with few observed events.

c. In the CSPS2 study there was a significant difference between aspirin and cilostazol in bleeding complications. In CAIST, CASISP, and PICASSO trials there was no significant difference between aspirin and cilostazol. However, the risk ratio reductions are similar.

***Abbreviations:*** *CI: confidence interval; RR: risk ratio*

**S3/B Table: summarizes the certainty of the cilostazol plus aspirin vs. aspirin studies:**

| **Certainty assessment** | | | | | | | **№ of patients** | | **Effect** | | **Certainty** | **Importance** |
| --- | --- | --- | --- | --- | --- | --- | --- | --- | --- | --- | --- | --- |
| **№ of studies** | **Study design** | **Risk of bias** | **Inconsistency** | **Indirectness** | **Imprecision** | **Other considerations** | **cilostazol + aspirin** | **aspirin** | **Relative (95% CI)** | **Absolute (95% CI)** |  |  |
| **Stroke** | | | | | | | | | | | | |
| 4 | randomized trials | not serious | not serious | not serious | very serious^a^ | none | 7/288 (2.4%) | 12/289 (4.2%) | **RR 1.70** (0.70 to 4.12) | **29 more per 1,000** (from 12 fewer to 130 more) | **⨁⨁◯◯ Low** |  |
| **Mortality** | | | | | | | | | | | | |
| 2 | randomized trials | not serious | not serious | not serious | very serious^b^ | none | 1/150 (0.7%) | 1/148 (0.7%) | not estimable |  | **⨁⨁◯◯ Low** |  |

**S3/B Table legend:** For the cilostazol plus aspirin vs. aspirin trials, the assessment found low certainty, because the total number of patients involved in these 4 studies was small, and the number of the observed endpoints was low.

a. The total number of patients involved in these 4 studies was small and the observed endpoint was low.

b. The total number of patients involved in these 2 studies was small and the observed endpoint was low.

***Abbreviations:*** *CI: confidence interval; RR: risk ratio*

# S4 Table: Individual and network meta-analysis results of the risk of stroke:

|  | ASA | ASA+  CILOSTAZOL | ASA+  CLOPIDOGREL | ASA+  DIPYRIDAMOLE | ASA+  SULFINPYRAZONE | ASA+  TICAGRELOR | CILOSTAZOL | CLOPIDOGREL | DABIGATRAN | DIPYRIDAMOLE | PENTOXIFYLLINE |
| --- | --- | --- | --- | --- | --- | --- | --- | --- | --- | --- | --- |
| ASA | **ASA** | 1.70  (0.70; 4.12) | **1.29**  **(1.17; 1.43)** | **1.16**  **(1.00; 1.35)** | 1.59  (0.85; 2.99) | **1.23**  **(1.05; 1.43)** | **1.50**  **(1.23; 1.84)** | 1.08  (0.94; 1.25) | 1.17  (0.96; 1.42) | 0.98  (0.82; 1.17) | . |
| ASA+  CILOSTAZOL | 1.27  (0.68; 2.38) | **ASA+**  **CILOSTAZOL** | 1.33  (0.55; 3.25) | . | . | . | . | . | . | . | . |
| ASA+  CLOPIDOGREL | **1.26**  **(1.16; 1.37)** | 0.99  (0.53; 1.87) | **ASA+**  **CLOPIDOGREL** | . | . | 1.42  (0.83; 2.42) | . | 0.98  (0.85; 1.13) | . | . | . |
| ASA+  DIPYRIDAMOLE | **1.19**  **(1.09; 1.31)** | 0.94  (0.50; 1.77) | 0.94  (0.85; 1.06) | **ASA+**  **DIPYRIDAMOLE** | . | . | . | 1.02  (0.93; 1.11) | . | **0.75**  **(0.61; 0.91)** | 0.42  (0.04; 4.37) |
| ASA+  SULFINPYRAZONE | 1.42  (0.80; 2.52) | 1.12  (0.48; 2.62) | 1.13  (0.63; 2.01) | 1.20  (0.67; 2.13) | **ASA+**  **SULFINPYRAZONE** | . | . | . | . | . | . |
| ASA+  TICAGRELOR | **1.26**  **(1.09; 1.46)** | 1.00  (0.52; 1.90) | 1.00  (0.85; 1.18) | 1.06  (0.89; 1.26) | 0.89  (0.49; 1.60) | **ASA+**  **TICAGRELOR** | . | . | . | . | . |
| CILOSTAZOL | **1.52**  **(1.26; 1.82)** | 1.19  (0.62; 2.30) | 1.20  (0.98; 1.47) | **1.27**  **(1.04; 1.56)** | 1.06  (0.59; 1.94) | 1.20  (0.95; 1.52) | **CILOSTAZOL** | . | . | . | . |
| CLOPIDOGREL | **1.18**  **(1.09; 1.29)** | 0.93  (0.50; 1.76) | 0.94  (0.85; 1.04) | 1.00  (0.92; 1.08) | 0.83  (0.47; 1.48) | 0.94  (0.79; 1.11) | **0.78**  **(0.64; 0.96)** | **CLOPIDOGREL** | . | . | . |
| DABIGATRAN | 1.17  (0.96; 1.42) | 0.92  (0.48; 1.78) | 0.93  (0.75; 1.15) | 0.98  (0.79; 1.22) | 0.82  (0.45; 1.50) | 0.93  (0.73; 1.18) | 0.77  (0.59; 1.01) | 0.99  (0.80; 1.22) | **DABIGATRAN** | . | . |
| DIPYRIDAMOLE | 0.95  (0.82; 1.10) | 0.75  (0.39; 1.43) | **0.75**  **(0.64; 0.89)** | **0.80**  **(0.68; 0.94)** | 0.67  (0.37; 1.20) | **0.75**  **(0.61; 0.93)** | **0.63**  **(0.50; 0.79)** | **0.80**  **(0.68; 0.94)** | 0.81  (0.64; 1.04) | **DIPYRIDAMOLE** | . |
| PENTOXIFYLLINE | 0.50  (0.05; 5.22) | 0.39  (0.03; 4.47) | 0.39  (0.04; 4.14) | 0.42  (0.04; 4.37) | 0.35  (0.03; 3.92) | 0.39  (0.04; 4.15) | 0.33  (0.03; 3.47) | 0.42  (0.04; 4.40) | 0.42  (0.04; 4.50) | 0.52  (0.05; 5.51) | **PENTOXIFYLLINE** |
| PLACEBO | **0.83**  **(0.77; 0.89)** | 0.65  (0.35; 1.23) | **0.66**  **(0.59; 0.73)** | **0.70**  **(0.63; 0.77)** | 0.58  (0.33; 1.03) | **0.66**  **(0.56; 0.77)** | **0.55**  **(0.45; 0.66)** | **0.70**  **(0.63; 0.77)** | **0.71**  **(0.58; 0.87)** | 0.87  (0.75; 1.01) | 1.67  (0.16; 17.55) |
| RIVAROXABAN | 0.93  (0.75; 1.14) | 0.73  (0.38; 1.42) | **0.73**  **(0.58; 0.92)** | **0.78**  **(0.62; 0.98)** | 0.65  (0.35; 1.19) | **0.73**  **(0.57; 0.95)** | **0.61**  **(0.46; 0.81)** | **0.78**  **(0.62; 0.98)** | 0.79  (0.59; 1.05) | 0.97  (0.75; 1.26) | 1.86  (0.18; 19.80) |
| SARPOGRELATE | 1.11  (0.81; 1.52) | 0.87  (0.43; 1.76) | 0.88  (0.65; 1.19) | 0.93  (0.67; 1.29) | 0.78  (0.41; 1.50) | 0.88  (0.62; 1.24) | 0.73  (0.51; 1.05) | 0.94  (0.68; 1.29) | 0.95 (  0.65; 1.38) | 1.17  (0.82; 1.65) | 2.24  (0.21; 24.00) |
| SULFINPYRAZONE | 0.74  (0.49; 1.13) | 0.58  (0.27; 1.24) | **0.59**  **(0.38; 0.90)** | **0.62**  **(0.40; 0.96)** | **0.52**  **(0.29; 0.94)** | **0.59**  **(0.38; 0.92)** | **0.49**  **(0.31; 0.77)** | **0.62**  **(0.41; 0.96)** | 0.63  (0.40; 1.01) | 0.78  (0.50; 1.22) | 1.49  (0.14; 16.28) |
| TICAGRELOR | **1.15**  **(1.01; 1.31)** | 0.91  (0.48; 1.73) | 0.91  (0.78; 1.07) | 0.97  (0.82; 1.13) | 0.81  (0.45; 1.45) | 0.91  (0.75; 1.11) | **0.76**  **(0.61; 0.95)** | 0.97  (0.83; 1.14) | 0.98  (0.78; 1.24) | 1.21  (0.99; 1.48) | 2.32  (0.22; 24.47) |
| TICLOPIDINE | 1.13  (0.99; 1.29) | 0.89  (0.47; 1.70) | 0.90  (0.77; 1.05) | 0.95  (0.81; 1.11) | 0.79  (0.44; 1.43) | 0.90  (0.73; 1.09) | **0.75**  **(0.60; 0.94)** | 0.96  (0.82; 1.11) | 0.97  (0.77; 1.22) | 1.19  (0.98; 1.45) | 2.28  (0.22; 24.07) |
| ASA+  TICLOPIDINE | 0.84  (0.32; 2.22) | 0.66  (0.21; 2.10) | 0.67  (0.25; 1.76) | 0.71  (0.27; 1.87) | 0.59  (0.19; 1.82) | 0.67  (0.25; 1.77) | 0.56  (0.21; 1.49) | 0.71  (0.27; 1.88) | 0.72  (0.27; 1.93) | 0.89  (0.33; 2.36) | 1.70  (0.13; 21.60) |
| TINZAPARIN | 0.65  (0.37; 1.12) | 0.51  (0.22; 1.18) | **0.51**  **(0.29; 0.90)** | **0.54**  **(0.31; 0.95)** | 0.45  (0.21; 1.01) | **0.51**  **(0.29; 0.91)** | **0.43**  **(0.24; 0.76)** | **0.55**  **(0.31; 0.96)** | **0.55**  **(0.31; 0.99)** | 0.68  (0.38; 1.21) | 1.30  (0.12; 14.62) |
| TRIFLUSAL | 0.93  (0.48; 1.79) | 0.73  (0.29; 1.82) | 0.74  (0.38; 1.43) | 0.78  (0.40; 1.51) | 0.65  (0.27; 1.55) | 0.73  (0.38; 1.44) | 0.61  (0.31; 1.21) | 0.78  (0.40; 1.52) | 0.79  (0.40; 1.57) | 0.98  (0.50; 1.91) | 1.87  (0.16; 21.52) |
| WARFARIN | 1.19  (0.86; 1.66) | 0.94  (0.46; 1.92) | 0.95  (0.67; 1.33) | 1.00  (0.71; 1.41) | 0.84  (0.43; 1.62) | 0.94  (0.66; 1.36) | 0.79  (0.54; 1.15) | 1.01  (0.71; 1.42) | 1.02  (0.69; 1.50) | 1.25  (0.87; 1.81) | 2.40  (0.22; 25.86) |

**S4 table continued.**

|  | PLACEBO | RIVAROXABAN | SARPOGRELATE | SULFINPYRAZONE | TICAGRELOR | TICLOPIDINE | ASA+  TICLOPDINE | TINZAPARIN | TRIFLUSAL | WARFARIN |
| --- | --- | --- | --- | --- | --- | --- | --- | --- | --- | --- |
| ASA | **0.86**  **(0.80; 0.92)** | 0.93  (0.75; 1.14) | . | 0.83  (0.51; 1.35) | **1.15**  **(1.01; 1.31)** | 1.07  (0.91; 1.25) | . | 0.65  (0.37; 1.12) | 0.93  (0.48; 1.79) | 1.17  (0.84; 1.65) |
| ASA+  CILOSTAZOL | . | . | . | . | . | . | . | . | . | . |
| ASA+  CLOPIDOGREL | . | . | 0.88  (0.65; 1.19) | . | . | . | . | . | . | . |
| ASA+  DIPYRIDAMOLE | **0.62**  **(0.54; 0.71)** | . | . | . | . | . | . | . | . | 1.52  (0.26; 8.82) |
| ASA+  SULFINPYRAZONE | 0.67  (0.35; 1.27) | . | . | **0.52**  **(0.28; 0.94)** | . | . | . | . | . | . |
| ASA+  TICAGRELOR | . | . | . | . | . | . | . | . | . | . |
| CILOSTAZOL | **0.53**  **(0.34; 0.81)** | . | . | . | . | . | . | . | . | . |
| CLOPIDOGREL | . | . | . | . | . | 1.07  (0.69; 1.66) | . | . | . | . |
| DABIGATRAN | . | . | . | . | . | . | . | . | . | . |
| DIPYRIDAMOLE | 0.85  (0.72; 1.01) | . | . | . | . | . | . | . | . | . |
| PENTOXIFYLLINE | . | . | . | . | . | . | . | . | . | . |
| PLACEBO | **PLACEBO** | . | . | 0.77  (0.46; 1.30) | . | **1.65**  **(1.20; 2.27)** | . | . | . | . |
| RIVAROXABAN | 1.12  (0.90; 1.40) | **RIVAROXABAN** | . | . | . | . | . | . | . | . |
| SARPOGRELATE | 1.34  (0.97; 1.85) | 1.20  (0.82; 1.75) | **SARPOGRELATE** | . | . | . | . | . | . | . |
| SULFINPYRAZONE | 0.89  (0.59; 1.36) | 0.80  (0.50; 1.28) | 0.67  (0.39; 1.13) | **SULFINPYRAZONE** | . | . | . | . | . | . |
| TICAGRELOR | **1.39**  **(1.20; 1.61)** | 1.24  (0.97; 1.59) | 1.04  (0.74; 1.46) | **1.55**  **(1.00; 2.42)** | **TICAGRELOR** | . | . | . | . | . |
| TICLOPIDINE | **1.37**  **(1.18; 1.58)** | 1.22  (0.95; 1.57) | 1.02  (0.72; 1.44) | 1.53  (0.98; 2.38) | 0.98  (0.82; 1.19) | **TICLOPIDINE** | 0.74 (0.29; 1.94) | . | . | . |
| ASA+  TICLOPDINE | 1.02  (0.39; 2.68) | 0.91  (0.34; 2.45) | 0.76  (0.27; 2.10) | 1.14  (0.40; 3.27) | 0.73  (0.28; 1.94) | 0.74  (0.29; 1.94) | **ASA+**  **TICLOPIDINE** | . | . | . |
| TINZAPARIN | 0.78  (0.45; 1.36) | 0.70  (0.39; 1.26) | 0.58  (0.31; 1.10) | 0.87  (0.44; 1.75) | **0.56**  **(0.32; 0.99)** | 0.57  (0.32; 1.01) | 0.77  (0.25; 2.34) | **TINZAPARIN** | . | . |
| TRIFLUSAL | 1.12  (0.58; 2.17) | 1.00  (0.50; 2.00) | 0.84  (0.40; 1.73) | 1.25  (0.57; 2.73) | 0.81  (0.41; 1.58) | 0.82  (0.42; 1.60) | 1.10  (0.34; 3.55) | 1.43  (0.61; 3.38) | **TRIFLUSAL** | . |
| WARFARIN | **1.44**  **(1.03; 2.02)** | 1.29  (0.87; 1.91) | 1.07  (0.68; 1.70) | 1.61  (0.94; 2.75) | 1.04  (0.73; 1.48) | 1.05  (0.74; 1.51) | 1.42  (0.51; 3.94) | 1.84  (0.97; 3.51) | 1.28  (0.62; 2.68) | **WARFARIN** |

**S4 Table legend:** Results are risk ratios (95% confidence intervals) from the network meta-analysis between the column and row defining intervention. The upper right triangle displays only the pooled effect sizes of the direct comparisons available in the network, like one would attain them if a conventional meta-analysis for each comparison were performed. Here RR < 1 means that the column defined treatment is worse. As we do not have direct evidence for all comparisons, some fields in the upper triangle remain empty. The lower left triangle contains the network meta-analysis effect sizes for each comparison. Here RR < 1 means that the column defined treatment is better. Significant differences are set in bold. ***Abbreviation:*** *ASA:* *acetylsalicylic acid, aspirin*

# S5 Table: Results of the inconsistency analyses of the network:

| Design | Q | degree of freedom | p-value |
| --- | --- | --- | --- |
| Total | 43.76 | 41 | 0.3551 |
| Within designs | 27.25 | 25 | 0.3435 |
| Between designs | 16.51 | 16 | 0.4178 |
| Design-specific decomposition |  |  |  |
| *ASA vs. ASA + Cilostazol* | 0.36 | 3 | 0.9478 |
| *ASA vs. ASA + Clopidogrel* | 6.48 | 9 | 0.6915 |
| *ASA vs. ASA + Dipyridamole* | 6.14 | 2 | 0.0464* |
| *ASA vs. Cilostazol* | 0.28 | 3 | 0.9639 |
| *ASA vs. Placebo* | 7.76 | 6 | 0.2566 |
| *ASA vs. Ticlopidine* | 6.18 | 1 | 0.0129* |
| *Clopidogrel vs. Ticlopidine* | 0.06 | 1 | 0.8107 |
| Detached designs |  |  |  |
| *ASA vs. ASA + Cilostazol* | 15.68 | 15 | 0.4039 |
| *ASA vs. ASA + Clopidogrel* | 15.41 | 15 | 0.4223 |
| *ASA vs. ASA +* *Dipyridamole* | 13.81 | 15 | 0.5401 |
| *ASA vs. ASA +* *Ticagrelor* | 14.75 | 15 | 0.4695 |
| *ASA vs. Cilostazol* | 16.48 | 15 | 0.3512 |
| *ASA vs. Clopidogrel* | 14.29 | 15 | 0.5039 |
| *ASA vs. Placebo* | 12.58 | 15 | 0.6347 |
| *ASA vs. Sulfinpyrazone* | 16.43 | 15 | 0.3538 |
| *ASA vs. Ticlopidine* | 14.33 | 15 | 0.5006 |
| *ASA vs. Warfarin* | 16.28 | 15 | 0.3634 |
| *ASA + Cilostazol vs. ASA + Clopidogrel* | 15.68 | 15 | 0.4039 |
| *ASA + Clopidogrel vs. ASA+* *Ticagrelor* | 14.75 | 15 | 0.4695 |
| *ASA + Clopidogrel vs. Clopidogrel* | 15.93 | 15 | 0.3870 |
| *ASA + Dipyridamole vs. Clopidogrel* | 15.46 | 15 | 0.4185 |
| *ASA + Dipyridamole vs. Placebo* | 15.19 | 15 | 0.4378 |
| *ASA + Dipyridamole vs. Warfarin* | 16.28 | 15 | 0.3634 |
| *Cilostazol vs. Placebo* | 16.48 | 15 | 0.3512 |
| *Clopidogrel vs. Ticlopidine* | 16.20 | 15 | 0.3686 |
| *Dipyridamole vs. Placebo* | 16.20 | 15 | 0.3690 |
| *Placebo vs. Ticlopidine* | 14.82 | 15 | 0.4642 |
| *ASA vs. ASA + Dipyridamole vs. Dipyridamole vs. Placebo* | 14.41 | 13 | 0.3458 |
| *ASA vs. ASA+ Dipyridamole vs. Placebo* | 14.34 | 14 | 0.4250 |
| *ASA vs. ASA+* *Sulfinpyrazone vs. Placebo vs. Sulfinpyrazone* | 15.66 | 14 | 0.3346 |

**S5 Table legend:** Q statistics to assess homogeneity/consistency did not show signs of heterogeneity in the network. Design-specific decomposition of within-designs Q statistics showed homogenous results except in the case of ASA + DIP and ASA + TICLO combinations when compared to ASA (marked with an asterisk). However, between-designs Q statistic after detaching single designs showed homogenous results. Q statistic to assess consistency under the assumption of a full design-by-treatment interaction in the random-effects model showed no sign of inconsistency: Q:14.64, degree of freedom:16, p-value: 0.5509, tau. within 0.0507. ***Abbreviations:*** *ASA: acetylsalicylic acid, aspirin*

# S6 Table: Results of the patient population stratified analyses:

|  |  | **Recurrent stroke** | **Mortality** | **MACE** | **Major Bleeding** | |
| --- | --- | --- | --- | --- | --- | --- |
| **Full Model (58 RCTs)** | | | | | |  |
| **ASA vs.** | ASA + Cilostazol | 0.6 [0.49-0.73] | 0.96 [0.65-1.42] | 0.66 [0.52-0.83] | 0.82 [0.34-1.96] | |
|  | ASA + Clopidogrel | 0.78 [0.71-0.85] | 1.07 [0.91-1.25] | 0.8 [0.74-0.86] | 1.78 [1.49-2.12] | |
|  | ASA + Dipyridamole | 0.87 [0.79-0.95] | 0.99 [0.9-1.09] | 0.88 [0.81-0.94] | 0.93 [0.78-1.11] | |
|  | ASA + Sulfinpyrazone | 1.02 [0.69-1.52] | 0.99 [0.5-1.98] | NA | NA | |
|  | ASA + Ticagrelor | 0.78 [0.68-0.88] | 1.25 [0.93-1.67] | 0.77 [0.67-0.89] | 2.21 [1.5-3.26] | |
|  | ASA + Ticlopidine | 0.79 [0.67-0.93] | NA | NA | 1.1 [0.36-3.39] | |
|  | Cilostazol | 0.68 [0.56-0.81] | 0.94 [0.65-1.37] | 0.74 [0.59-0.93] | 0.37 [0.15-0.88] | |
|  | Clopidogrel | 0.88 [0.8-0.96] | 1.05 [0.93-1.18] | 0.9 [0.84-0.96] | 0.8 [0.66-0.96] | |
|  | Dabigatran | 0.86 [0.69-1.06] | 0.97 [0.67-1.39] | 0.89 [0.74-1.07] | 1.2 [0.87-1.67] | |
|  | Dipyridamole | 0.98 [0.86-1.12] | 0.97 [0.85-1.11] | 0.99 [0.89-1.1] | 0.42 [0.31-0.56] | |
|  | Pentoxifylline | 2.09 [0.2-21.97] | 0.35 [0.01-9.22] | NA | NA | |
|  | Placebo | 1.21 [1.12-1.31] | 1.1 [1.03-1.17] | 1.2 [1.1-1.3] | 0.54 [0.44-0.66] | |
|  | Rivaroxaban | 1.08 [0.86-1.36] | 1.25 [0.87-1.79] | 1.07 [0.88-1.3] | 2.69 [1.67-4.33] | |
|  | Sarpogrelate | 1.14 [0.82-1.57] | 1.46 [0.68-3.13] | 1.08 [0.82-1.42] | NA | |
|  | Sulfinpyrazone | 1.16 [0.78-1.71] | 0.97 [0.49-1.94] | 2.07 [0.39-10.87] | NA | |
|  | Ticagrelor | 0.88 [0.77-0.99] | 1.22 [0.92-1.63] | 0.87 [0.77-0.99] | 0.99 [0.68-1.45] | |
|  | Ticlopidine | 0.89 [0.77-1.03] | 0.93 [0.79-1.09] | 0.95 [0.81-1.13] | 0.49 [0.16-1.53] | |
|  | Tinzaparin | 1.55 [0.88-2.71] | 0.97 [0.75-1.26] | NA | 2.64 [0.77-9.01] | |
|  | Triflusal | 1.08 [0.56-2.09] | 0.47 [0.18-1.21] | 0.91 [0.55-1.49] | 0.14 [0.02-1.17] | |
|  | Warfarin | 0.84 [0.59-1.18] | 1.16 [0.84-1.61] | 1.06 [0.79-1.41] | 1.72 [1.18-2.5] | |
| **Minor stroke or TIA (19 RCTs)** | | | | | |  |
| **ASA vs.** | ASA + Cilostazol | 0.67 [0.12-3.77] | NA | NA | 0.33 [0.01-7.93] | |
|  | ASA + Clopidogrel | 0.76 [0.68-0.86] | 0.95 [0.64-1.42] | 0.76 [0.67-0.86] | 1.75 [1.03-2.97] | |
|  | ASA + Dipyridamole | 1.3 [0.22-7.76] | 0.88 [0.67-1.14] | 0.78 [0.64-0.96] | 0.7 [0.47-1.05] | |
|  | ASA + Ticagrelor | 0.79 [0.68-0.91] | 1.33 [0.83-2.16] | 0.53 [0.31-0.9] | 3 [1.63-5.52] | |
|  | Dabigatran | 0.86 [0.7-1.04] | 0.97 [0.67-1.39] | 0.89 [0.75-1.07] | 1.2 [0.87-1.67] | |
|  | Placebo | 1.15 [0.98-1.34] | 1.1 [0.93-1.31] | 1.17 [1.04-1.31] | NA | |
|  | Rivaroxaban | 1.08 [0.87-1.34] | 1.25 [0.87-1.79] | 1.07 [0.88-1.29] | 2.69 [1.67-4.33] | |
|  | Ticagrelor | 0.87 [0.76-0.99] | 1.18 [0.83-1.67] | 0.89 [0.79-1.01] | 0.82 [0.51-1.31] | |
|  | Ticlopidine | 0.82 [0.68-0.99] | 0.9 [0.74-1.09] | NA | NA | |
|  | Triflusal | 1.08 [0.56-2.08] | 0.47 [0.18-1.21] | 0.91 [0.55-1.49] | 0.14 [0.02-1.17] | |
|  | Warfarin | 0.85 [0.61-1.2] | 2.25 [1.22-4.13] | 1.06 [0.79-1.41] | 2.23 [1.15-4.31] | |
| **TIA (5 RCTs)** | | | | | |  |
| **ASA vs.** | ASA + Dipyridamole | 0.87 [0.51-1.48] | 1.19 [0.79-1.8] | 0.96 [0.74-1.25] | NA | |
|  | ASA + Sulfinpyrazone | 0.7 [0.34-1.43] | 1.44 [0.47-4.42] | NA | NA | |
|  | Pentoxifylline | 2.09 [0.18-24.1] | 0.42 [0.02-11.4] | NA | NA | |
|  | Placebo | 1.18 [0.65-2.14] | 2.57 [1.05-6.29] | NA | NA | |
|  | Sulfinpyrazone | 1.33 [0.73-2.42] | 1.95 [0.73-5.21] | 2.07 [0.39-10.87] | NA | |
| **Definite stroke (TIA excluded) (53 RCTs)** | | | | | |  |
| **ASA vs.** | ASA + Cilostazol | 0.6 [0.5-0.74] | 0.97 [0.65-1.42] | 0.66 [0.52-0.83] | 0.82 [0.34-1.96] | |
|  | ASA + Clopidogrel | 0.78 [0.71-0.86] | 1.06 [0.91-1.24] | 0.79 [0.73-0.86] | 1.78 [1.49-2.12] | |
|  | ASA + Dipyridamole | 0.87 [0.79-0.96] | 0.98 [0.88-1.08] | 0.87 [0.8-0.94] | 0.93 [0.78-1.11] | |
|  | ASA + Ticagrelor | 0.78 [0.69-0.89] | 1.25 [0.94-1.67] | 0.77 [0.66-0.89] | 2.21 [1.5-3.26] | |
|  | ASA + Ticlopidine | 0.8 [0.67-0.94] | NA | NA | 1.1 [0.36-3.39] | |
|  | Cilostazol | 0.67 [0.56-0.81] | 0.94 [0.65-1.37] | 0.74 [0.59-0.93] | 0.37 [0.15-0.88] | |
|  | Clopidogrel | 0.87 [0.8-0.96] | 1.04 [0.92-1.17] | 0.9 [0.83-0.97] | 0.8 [0.66-0.96] | |
|  | Dabigatran | 0.86 [0.69-1.06] | 0.97 [0.67-1.39] | 0.89 [0.74-1.08] | 1.2 [0.87-1.67] | |
|  | Dipyridamole | 0.97 [0.85-1.11] | 0.95 [0.83-1.1] | 0.98 [0.88-1.1] | 0.42 [0.31-0.56] | |
|  | Placebo | 1.21 [1.12-1.31] | 1.09 [1.03-1.16] | 1.19 [1.1-1.3] | 0.54 [0.44-0.66] | |
|  | Rivaroxaban | 1.08 [0.86-1.36] | 1.25 [0.87-1.79] | 1.07 [0.87-1.3] | 2.69 [1.67-4.33] | |
|  | Sarpogrelate | 1.14 [0.83-1.56] | 1.46 [0.68-3.13] | 1.08 [0.81-1.42] | NA | |
|  | Ticagrelor | 0.87 [0.77-0.99] | 1.22 [0.92-1.62] | 0.87 [0.77-0.99] | 0.99 [0.68-1.45] | |
|  | Ticlopidine | 0.89 [0.77-1.03] | 0.93 [0.79-1.09] | 0.95 [0.8-1.13] | 0.49 [0.16-1.53] | |
|  | Tinzaparin | 1.55 [0.88-2.71] | 0.97 [0.75-1.26] | NA | 2.64 [0.77-9.01] | |
|  | Triflusal | 1.08 [0.56-2.09] | 0.47 [0.18-1.21] | 0.91 [0.55-1.49] | 0.14 [0.02-1.17] | |
|  | Warfarin | 0.84 [0.59-1.18] | 1.16 [0.84-1.61] | 1.06 [0.79-1.41] | 1.72 [1.18-2.5] | |

**S6 Table Legend:** The table presents the risk ratio and 95% confidence intervals of aspirin monotherapy compared to the other treatments available in the respective network. Stratification was done based on the main inclusion criteria of the trials. ***Abbreviations:*** *ASA:* *aspirin, acetylsalicylic acid, DOAC: direct acting oral anticoagulant, MACE: major cardiovascular adverse events, NA: not applicable, RCTs: randomized controlled trials, TIA: transient ischemic attack*

# S7 Table: Results of the treatment protocol stratified subgroup analyses:

|  |  | **Recurrent stroke** | **Mortality** | **MACE** | **Major Bleeding** |  |
| --- | --- | --- | --- | --- | --- | --- |
| **Full Model (58 RCTs)** | | | | | |  |
| **ASA vs.** | ASA + Cilostazol | 0.6 [0.49-0.73] | 0.96 [0.65-1.42] | 0.66 [0.52-0.83] | 0.82 [0.34-1.96] |  |
|  | ASA + Clopidogrel | 0.78 [0.71-0.85] | 1.07 [0.91-1.25] | 0.8 [0.74-0.86] | 1.78 [1.49-2.12] |  |
|  | ASA + Dipyridamole | 0.87 [0.79-0.95] | 0.99 [0.9-1.09] | 0.88 [0.81-0.94] | 0.93 [0.78-1.11] |  |
|  | ASA + Sulfinpyrazone | 1.02 [0.69-1.52] | 0.99 [0.5-1.98] | NA | NA |  |
|  | ASA + Ticagrelor | 0.78 [0.68-0.88] | 1.25 [0.93-1.67] | 0.77 [0.67-0.89] | 2.21 [1.5-3.26] |  |
|  | ASA + Ticlopidine | 0.79 [0.67-0.93] | NA | NA | 1.1 [0.36-3.39] |  |
|  | Cilostazol | 0.68 [0.56-0.81] | 0.94 [0.65-1.37] | 0.74 [0.59-0.93] | 0.37 [0.15-0.88] |  |
|  | Clopidogrel | 0.88 [0.8-0.96] | 1.05 [0.93-1.18] | 0.9 [0.84-0.96] | 0.8 [0.66-0.96] |  |
|  | Dabigatran | 0.86 [0.69-1.06] | 0.97 [0.67-1.39] | 0.89 [0.74-1.07] | 1.2 [0.87-1.67] |  |
|  | Dipyridamole | 0.98 [0.86-1.12] | 0.97 [0.85-1.11] | 0.99 [0.89-1.1] | 0.42 [0.31-0.56] |  |
|  | Pentoxifylline | 2.09 [0.2-21.97] | 0.35 [0.01-9.22] | NA | NA |  |
|  | Placebo | 1.21 [1.12-1.31] | 1.1 [1.03-1.17] | 1.2 [1.1-1.3] | 0.54 [0.44-0.66] |  |
|  | Rivaroxaban | 1.08 [0.86-1.36] | 1.25 [0.87-1.79] | 1.07 [0.88-1.3] | 2.69 [1.67-4.33] |  |
|  | Sarpogrelate | 1.14 [0.82-1.57] | 1.46 [0.68-3.13] | 1.08 [0.82-1.42] | NA |  |
|  | Sulfinpyrazone | 1.16 [0.78-1.71] | 0.97 [0.49-1.94] | 2.07 [0.39-10.87] | NA |  |
|  | Ticagrelor | 0.88 [0.77-0.99] | 1.22 [0.92-1.63] | 0.87 [0.77-0.99] | 0.99 [0.68-1.45] |  |
|  | Ticlopidine | 0.89 [0.77-1.03] | 0.93 [0.79-1.09] | 0.95 [0.81-1.13] | 0.49 [0.16-1.53] |  |
|  | Tinzaparin | 1.55 [0.88-2.71] | 0.97 [0.75-1.26] | NA | 2.64 [0.77-9.01] |  |
|  | Triflusal | 1.08 [0.56-2.09] | 0.47 [0.18-1.21] | 0.91 [0.55-1.49] | 0.14 [0.02-1.17] |  |
|  | Warfarin | 0.84 [0.59-1.18] | 1.16 [0.84-1.61] | 1.06 [0.79-1.41] | 1.72 [1.18-2.5] |  |
| **Acute studies (14 RCTs)** | | | | | |  |
| **ASA vs.** | ASA + Cilostazol | 0.54 [0.21-1.36] | NA | NA | 1.01 [0.21-4.94] |  |
|  | ASA + Clopidogrel | 0.76 [0.67-0.85] | 0.99 [0.67-1.47] | 0.76 [0.67-0.86] | 1.89 [1.13-3.18] |  |
|  | ASA + Dipyridamole | 0.49 [0.2-1.22] | 4.84 [0.22-105.92] | 0.63 [0.3-1.34] | 1 [0.02-50.66] |  |
|  | ASA + Ticagrelor | 0.79 [0.68-0.91] | 1.34 [0.82-2.18] | 0.53 [0.31-0.9] | 2.91 [1.56-5.43] |  |
|  | Cilostazol | 0.59 [0.24-1.48] | NA | 0.66 [0.24-1.81] | 0.29 [0.07-1.27] |  |
|  | Placebo | 1.15 [1.04-1.27] | 1.08 [0.98-1.2] | NA | 0.59 [0.45-0.78] |  |
|  | Ticagrelor | 0.87 [0.76-0.99] | 1.18 [0.82-1.68] | 0.89 [0.79-1.01] | 0.84 [0.51-1.4] |  |
|  | Tinzaparin | 1.55 [0.89-2.68] | 0.97 [0.74-1.27] | NA | 2.64 [0.76-9.16] | |
| **Acute studies with less than 30 days of follow-up (6 RCTs)** | | | | | |  |
| **ASA vs.** | ASA + Clopidogrel | 0.55 [0.31-0.95] | 1.83 [0.36-9.33] | 0.66 [0.19-2.31] | 3.01 [0.71-12.79] |  |
|  | ASA + Dipyridamole | 0.49 [0.2-1.23] | 4.84 [0.22-105.98] | 0.63 [0.3-1.34] | 1 [0.02-50.39] |  |
|  | ASA + Ticagrelor | 0.81 [0.66-1] | 1.33 [0.8-2.2] | NA | 3.25 [1.66-6.39] |  |
|  | Placebo | 1.15 [1-1.32] | 1.09 [0.98-1.21] | NA | 0.59 [0.47-0.74] |  |
|  | Tinzaparin | 1.55 [0.87-2.73] | 0.97 [0.73-1.28] | NA | 2.64 [0.77-9.01] |  |
| **Acute studies with less than 90 days of follow-up (11 RCTs)** | | | | | |  |
| **ASA vs.** | ASA + Clopidogrel | 0.74 [0.6-0.93] | 1.46 [0.78-2.73] | 0.75 [0.61-0.93] | 2.62 [1.46-4.73] |  |
|  | ASA + Dipyridamole | 0.49 [0.2-1.24] | 4.84 [0.22-105.8] | 0.63 [0.3-1.34] | 1 [0.02-50.39] |  |
|  | ASA + Ticagrelor | 0.77 [0.62-0.94] | 1.38 [0.85-2.23] | 0.52 [0.3-0.92] | 3.27 [1.77-6.02] |  |
|  | Cilostazol | 0.55 [0.18-1.62] | NA | 0.66 [0.24-1.81] | 0.39 [0.08-2.01] |  |
|  | Placebo | 1.15 [0.99-1.34] | 1.08 [1-1.16] | NA | 0.59 [0.47-0.74] |  |
|  | Ticagrelor | 0.87 [0.71-1.07] | 1.18 [0.83-1.67] | 0.89 [0.79-1.01] | 0.82 [0.51-1.31] |  |
|  | Tinzaparin | 1.55 [0.87-2.75] | 0.97 [0.75-1.26] | NA | 2.64 [0.77-9.01] |  |
| **Chronic studies (44 RCTs)** | | | | | |  |
| **ASA vs.** | ASA + Cilostazol | 0.61 [0.49-0.76] | 0.97 [0.65-1.44] | 0.67 [0.52-0.87] | 0.88 [0.3-2.59] |  |
|  | ASA + Clopidogrel | 0.8 [0.7-0.92] | 1.11 [0.92-1.34] | 0.82 [0.73-0.92] | 1.73 [1.43-2.1] |  |
|  | ASA + Dipyridamole | 0.9 [0.8-1] | 1.01 [0.9-1.13] | 0.88 [0.8-0.97] | 0.91 [0.75-1.09] |  |
|  | ASA + Sulfinpyrazone | 1.04 [0.69-1.56] | 1.02 [0.51-2.05] | NA | NA |  |
|  | ASA + Ticlopidine | 0.8 [0.66-0.96] | NA | NA | 1.07 [0.35-3.31] |  |
|  | Cilostazol | 0.68 [0.56-0.83] | 0.95 [0.65-1.39] | 0.75 [0.59-0.96] | NA |  |
|  | Clopidogrel | 0.91 [0.81-1.02] | 1.09 [0.94-1.27] | 0.91 [0.83-1.01] | 0.79 [0.64-0.96] |  |
|  | Dabigatran | 0.86 [0.67-1.09] | 0.97 [0.67-1.4] | 0.89 [0.72-1.1] | 1.2 [0.87-1.67] |  |
|  | Dipyridamole | 1.01 [0.87-1.18] | 0.99 [0.85-1.16] | 0.98 [0.86-1.12] | 0.41 [0.3-0.56] |  |
|  | Pentoxifylline | 2.15 [0.2-22.71] | 0.36 [0.01-9.44] | NA | NA |  |
|  | Placebo | 1.26 [1.13-1.41] | 1.15 [1.03-1.29] | 1.2 [1.09-1.32] | 0.4 [0.26-0.61] |  |
|  | Rivaroxaban | 1.08 [0.84-1.39] | 1.25 [0.86-1.81] | 1.07 [0.86-1.33] | 2.69 [1.67-4.33] |  |
|  | Sarpogrelate | 1.14 [0.81-1.59] | 1.46 [0.68-3.15] | 1.08 [0.8-1.44] | NA |  |
|  | Sulfinpyrazone | 1.17 [0.79-1.75] | 1.01 [0.5-2.01] | 2.07 [0.39-10.91] | NA |  |
|  | Ticlopidine | 0.9 [0.77-1.05] | 0.94 [0.79-1.11] | 0.95 [0.8-1.14] | 0.49 [0.16-1.5] |  |
|  | Triflusal | 1.08 [0.55-2.11] | 0.47 [0.18-1.21] | 0.91 [0.55-1.51] | 0.14 [0.02-1.17] |  |
|  | Warfarin | 0.84 [0.59-1.2] | 1.17 [0.84-1.63] | 1.06 [0.78-1.44] | 1.72 [1.18-2.5] |  |
| **Antiplatelet studies (52 RCTs)** | | | | | |  |
| **ASA vs.** | ASA + Cilostazol | 0.6 [0.49-0.73] | 0.96 [0.65-1.42] | 0.66 [0.52-0.83] | 0.82 [0.34-1.96] | |
|  | ASA + Clopidogrel | 0.78 [0.71-0.85] | 1.07 [0.92-1.25] | 0.8 [0.74-0.86] | 1.78 [1.49-2.13] | |
|  | ASA + Dipyridamole | 0.87 [0.79-0.96] | 0.99 [0.9-1.09] | 0.88 [0.81-0.94] | 0.93 [0.78-1.12] | |
|  | ASA + Sulfinpyrazone | 1.02 [0.69-1.52] | 0.99 [0.5-1.98] | NA | NA | |
|  | ASA + Ticagrelor | 0.77 [0.68-0.88] | 1.25 [0.93-1.67] | 0.77 [0.67-0.89] | 2.21 [1.5-3.26] | |
|  | ASA + Ticlopidine | 0.79 [0.67-0.93] | NA | NA | 1.1 [0.36-3.4] | |
|  | Cilostazol | 0.68 [0.56-0.81] | 0.94 [0.65-1.37] | 0.74 [0.59-0.93] | 0.37 [0.15-0.88] | |
|  | Clopidogrel | 0.88 [0.8-0.96] | 1.05 [0.93-1.19] | 0.9 [0.84-0.96] | 0.8 [0.66-0.97] | |
|  | Dipyridamole | 0.98 [0.86-1.12] | 0.97 [0.85-1.11] | 0.99 [0.89-1.1] | 0.42 [0.31-0.56] | |
|  | Pentoxifylline | 2.08 [0.2-21.95] | 0.35 [0.01-9.24] | NA | NA | |
|  | Placebo | 1.21 [1.12-1.31] | 1.1 [1.03-1.17] | 1.2 [1.1-1.3] | 0.54 [0.44-0.66] | |
|  | Sarpogrelate | 1.14 [0.82-1.57] | 1.46 [0.68-3.13] | 1.08 [0.82-1.42] | NA | |
|  | Sulfinpyrazone | 1.16 [0.78-1.71] | 0.97 [0.49-1.94] | 2.07 [0.39-10.87] | NA | |
|  | Ticagrelor | 0.88 [0.77-1] | 1.22 [0.92-1.63] | 0.87 [0.77-0.99] | 0.99 [0.68-1.45] | |
|  | Ticlopidine | 0.89 [0.77-1.03] | 0.93 [0.79-1.09] | 0.95 [0.81-1.13] | 0.5 [0.16-1.53] | |
|  | Triflusal | 1.08 [0.55-2.09] | 0.47 [0.18-1.21] | 0.91 [0.55-1.49] | 0.14 [0.02-1.17] | |

**S7 Table Legend:** The table presents the risk ratio and 95% confidence intervals of aspirin monotherapy compared to the other treatments available in the respective network. Stratification was done based on the pharmacological and the follow-up protocol of the included trials. Antiplatelet studies were defined with the exclusion of trials testing anticoagulants. Studies were considered acute if patients were enrolled within 48 hours of the stroke event. According to the follow-up length, further subgroups were defined with follow-up limited to one month or three months or unlimited. ***Abbreviations:*** *ASA: aspirin, acetylsalicylic acid, DOAC: direct acting oral anticoagulant, MACE: major cardiovascular adverse events, NA: not applicable, RCTs: randomized controlled trials, TIA: transient ischemic attack*

# Citations of the included trials:

1. Johnston SC, Amarenco P, Denison H, Evans SR, Himmelmann A, James S, et al. Ticagrelor and Aspirin or Aspirin Alone in Acute Ischemic Stroke or TIA. N Engl J Med. 2020;383(3):207–17.

2. Johnston SC, Amarenco P, Albers GW, Denison H, Easton JD, Evans SR, et al. Ticagrelor versus aspirin in acute stroke or transient ischemic attack. N Engl J Med. 2016 Jul;375(1):35–43.

3. Wang Y, Chen W, Lin Y, Meng X, Chen G, Wang Z, et al. Ticagrelor plus aspirin versus clopidogrel plus aspirin for platelet reactivity in patients with minor stroke or transient ischaemic attack: Open label, blinded endpoint, randomised controlled phase II trial. BMJ [Internet]. 2019 Jun 6 [cited 2020 Sep 17];365. Available from: http://dx.doi.org/10.1136/bmj.l2211

4. Benavente OR, Hart RG, McClure LA, Szychowski JM, Coffey CS, Pearce LA. Effects of Clopidogrel Added to Aspirin in Patients with Recent Lacunar Stroke. N Engl J Med [Internet]. 2012 Aug 30 [cited 2020 Sep 17];367(9):817–25. Available from: http://www.nejm.org/doi/10.1056/NEJMoa1204133

5. Gent M. A randomised, blinded, trial of clopidogrel versus aspirin in patients at risk of ischaemic events (CAPRIE). Lancet [Internet]. 1996 Nov 16 [cited 2020 Sep 17];348(9038):1329–39. Available from: http://www.thelancet.com/article/S0140673696094573/fulltext

6. Chen ZM. CAST: Randomised placebo-controlled trial of early aspirin use in 20,000 patients with acute ischaemic stroke. Lancet [Internet]. 1997 Jun 7 [cited 2020 Sep 17];349(9066):1641–9. Available from: http://www.thelancet.com/article/S0140673697040105/fulltext

7. Diener HC, Cunha L, Forbes C, Sivenius J, Smets P, Lowenthal A. European stroke prevention study 2. Dipyridamole and acetylsalicylic acid in the secondary prevention of stroke. J Neurol Sci [Internet]. 1996 Nov 1 [cited 2020 Sep 17];143(1–2):1–13. Available from: http://www.jns-journal.com/article/S0022510X96003085/fulltext

8. Lowenthal A. European stroke prevention study. Stroke [Internet]. 1990 Aug [cited 2020 Sep 17];21(8):1122–30. Available from: https://www.ahajournals.org/doi/10.1161/01.STR.21.8.1122

9. Bousser MG, Eschwege E, Haguenau M, Lefaucconnier JM, Thibult N, Touboul D, et al. “AICLA” controlled trial of aspirin and dipyridamole in the secondary prevention of athero-thrombotic cerebral ischemia. Stroke [Internet]. 1983 Jan [cited 2020 Sep 17];14(1):5–14. Available from: https://www.ahajournals.org/doi/10.1161/01.STR.14.1.5

10. Diener PHC, Bogousslavsky PJ, Brass PLM, Cimminiello PC, Csiba PL, Kaste PM, et al. Aspirin and clopidogrel compared with clopidogrel alone after recent ischaemic stroke or transient ischaemic attack in high-risk patients (MATCH): Randomised, double-blind, placebo-controlled trial. Lancet [Internet]. 2004 Jul 24 [cited 2020 Sep 17];364(9431):331–7. Available from: http://www.thelancet.com/article/S0140673604167214/fulltext

11. Sacco RL, Diener H-C, Yusuf S, Cotton D, Ôunpuu S, Lawton WA, et al. Aspirin and Extended-Release Dipyridamole versus Clopidogrel for Recurrent Stroke. N Engl J Med [Internet]. 2008 Sep 18 [cited 2020 Sep 17];359(12):1238–51. Available from: http://www.nejm.org/doi/abs/10.1056/NEJMoa0805002

12. Chimowitz MI, Lynn MJ, Howlett-Smith H, Stern BJ, Hertzberg VS, Frankel MR, et al. Comparison of Warfarin and Aspirin for Symptomatic Intracranial Arterial Stenosis. N Engl J Med [Internet]. 2005 Mar 31 [cited 2020 Sep 17];352(13):1305–16. Available from: http://www.nejm.org/doi/abs/10.1056/NEJMoa043033

13. Halkes PHA. Aspirin plus dipyridamole versus aspirin alone after cerebral ischaemia of arterial origin (ESPRIT): randomised controlled trial. Lancet [Internet]. 2006 May 20 [cited 2020 Sep 17];367(9523):1665–73. Available from: http://www.thelancet.com/article/S0140673606687345/fulltext

14. Gent M, Donald Easton J, Hachinski VC, Panak E, Sicurella J, Blakely JA, et al. THE CANADIAN AMERICAN TICLOPIDINE STUDY (CATS) IN THROMBOEMBOLIC STROKE. Lancet [Internet]. 1989 Jun 3 [cited 2020 Sep 17];333(8649):1215–20. Available from: http://www.thelancet.com/article/S0140673689923271/fulltext

15. Gorelick PB, Richardson DJ, Kelly M, Ruland S, Hung E, Harris Y, et al. Aspirin and Ticlopidine for Prevention of Recurrent Stroke in Black Patients: A Randomized Trial. J Am Med Assoc [Internet]. 2003 Jun 11 [cited 2020 Sep 17];289(22):2947–57. Available from: https://jamanetwork.com/

16. Gotoh F, Tohgi H, Hirai S, Terashi A, Fukuuchi Y, Otomo E, et al. Cilostazol stroke prevention study: A placebo-controlled double-blind trial for secondary prevention of cerebral infarction. J Stroke Cerebrovasc Dis [Internet]. 2000 Jul 1 [cited 2020 Sep 17];9(4):147–57. Available from: http://www.strokejournal.org/article/S1052305700700292/fulltext

17. Hass WK, Easton JD, Adams HP, Pryse-Phillips W, Molony BA, Anderson S, et al. A Randomized Trial Comparing Ticlopidine Hydrochloride with Aspirin for the Prevention of Stroke in High-Risk Patients. N Engl J Med [Internet]. 1989 Aug 24 [cited 2020 Sep 17];321(8):501–7. Available from: http://www.nejm.org/doi/abs/10.1056/NEJM198908243210804

18. Fields WS, Lemak NA, Frankowski RF, Hardy RJ. Controlled trial of aspirin in cerebral ischemia. Stroke [Internet]. 1977 May [cited 2020 Sep 17];8(3):301–14. Available from: https://www.ahajournals.org/doi/10.1161/01.STR.8.3.301

19. Herskovits E, Famulari A, Tamaroff L, Gonzalez AM, Vazquez A, Smud R, et al. RANDOMISED TRIAL OF PENTOXIFYLLINE VERSUS ACETYLSALICYLIC ACID PLUS DIPYRIDAMOLE IN PREVENTING TRANSIENT ISCHAEMIC ATTACKS. Lancet [Internet]. 1981 May 2 [cited 2020 Sep 17];317(8227):966–8. Available from: http://www.thelancet.com/article/S0140673681917323/fulltext

20. Hong KS, Lee SH, Kim EG, Cho KH, Chang D Il, Rha JH, et al. Recurrent Ischemic Lesions after Acute Atherothrombotic Stroke: Clopidogrel Plus Aspirin Versus Aspirin Alone. Stroke [Internet]. 2016 Sep 1 [cited 2020 Sep 17];47(9):2323–30. Available from: https://www.ahajournals.org/doi/10.1161/STROKEAHA.115.012293

21. Huang Y, Cheng Y, Wu J, Li Y, Xu E, Hong Z, et al. Cilostazol as an alternative to aspirin after ischaemic stroke: a randomised, double-blind, pilot study. Lancet Neurol [Internet]. 2008 Jun 1 [cited 2020 Sep 17];7(6):494–9. Available from: http://www.thelancet.com/article/S1474442208700942/fulltext

22. Sandercock PAG. The International Stroke Trial (IST): A randomised trial of aspirin, subcutaneous heparin, both, or neither among 19 435 patients with acute ischaemic stroke. Lancet [Internet]. 1997 May 31 [cited 2020 Sep 17];349(9065):1569–81. Available from: http://www.thelancet.com/article/S0140673697040117/fulltext

23. Claiborne Johnston S, Donald Easton J, Farrant M, Barsan W, Conwit RA, Elm JJ, et al. Clopidogrel and aspirin in acute ischemic stroke and high-risk TIA. N Engl J Med [Internet]. 2018 Jul 19 [cited 2020 Sep 14];379(3):215–25. Available from: https://pubmed.ncbi.nlm.nih.gov/29766750/

24. Kennedy J, Hill MD, Ryckborst KJ, Eliasziw M, Demchuk AM, Buchan AM. Fast assessment of stroke and transient ischaemic attack to prevent early recurrence (FASTER): a randomised controlled pilot trial. Lancet Neurol [Internet]. 2007 Nov 1 [cited 2020 Sep 17];6(11):961–9. Available from: http://neurology.thelancet.com

25. Kim BJ, Lee EJ, Kwon SU, Park JH, Kim YJ, Hong KS, et al. Prevention of cardiovascular events in Asian patients with ischaemic stroke at high risk of cerebral haemorrhage (PICASSO): a multicentre, randomised controlled trial. Lancet Neurol [Internet]. 2018 Jun 1 [cited 2020 Sep 17];17(6):509–18. Available from: www.thelancet.com/neurology

26. Lee Y-S, Bae H-J, Kang D-W, Lee S-H, Yu K, Park J-M, et al. Cilostazol in Acute Ischemic Stroke Treatment (CAIST Trial): A Randomized Double-Blind Non-Inferiority Trial. Cerebrovasc Dis [Internet]. 2011 Jul [cited 2020 Sep 17];32(1):65–71. Available from: https://www.karger.com/Article/FullText/327036

27. Dengler R, Diener HC, Schwartz A, Grond M, Schumacher H, Machnig T, et al. Early treatment with aspirin plus extended-release dipyridamole for transient ischaemic attack or ischaemic stroke within 24 h of symptom onset (EARLY trial): a randomised, open-label, blinded-endpoint trial. Lancet Neurol [Internet]. 2010 Feb 1 [cited 2020 Sep 17];9(2):159–66. Available from: http://www.dgn.

28. Kwon SU, Cho YJ, Koo JS, Bae HJ, Lee YS, Hong KS, et al. Cilostazol prevents the progression of the symptomatic intracranial arterial stenosis: The multicenter double-blind placebo-controlled trial of cilostazol in symptomatic intracranial arterial stenosis. Stroke [Internet]. 2005 Apr 1 [cited 2020 Sep 17];36(4):782–6. Available from: https://www.ahajournals.org/doi/10.1161/01.STR.0000157667.06542.b7

29. Mohr JP, Thompson JLP, Lazar RM, Levin B, Sacco RL, Furie KL, et al. A Comparison of Warfarin and Aspirin for the Prevention of Recurrent Ischemic Stroke. N Engl J Med [Internet]. 2001 Nov 15 [cited 2020 Sep 17];345(20):1444–51. Available from: http://www.nejm.org/doi/abs/10.1056/NEJMoa011258

30. The Salt Collaborative Group. Swedish Aspirin Low-dose Trial (SALT) of 75 mg aspirin as secondary prophylaxis after cerebrovascular ischaemic events. Lancet [Internet]. 1991 Nov 30 [cited 2020 Sep 17];338(8779):1345–9. Available from: http://www.thelancet.com/article/014067369192233R/fulltext

31. Nakamura T, Tsuruta S, Uchiyama S. Cilostazol combined with aspirin prevents early neurological deterioration in patients with acute ischemic stroke: A pilot study. J Neurol Sci [Internet]. 2012 Feb 15 [cited 2020 Sep 17];313(1–2):22–6. Available from: http://www.jns-journal.com/article/S0022510X11006009/fulltext

32. Shinohara Y, Katayama Y, Uchiyama S, Yamaguchi T, Handa S, Matsuoka K, et al. Cilostazol for prevention of secondary stroke (CSPS 2): An aspirin-controlled, double-blind, randomised non-inferiority trial. Lancet Neurol [Internet]. 2010 Oct 1 [cited 2020 Sep 17];9(10):959–68. Available from: http://www.thelancet.com/article/S1474442210701988/fulltext

33. Hankey GJ, Johnston SC, Easton JD, Hacke W, Mas JL, Brennan D, et al. Effect of clopidogrel plus ASA vs. ASA early after TIA and ischaemic stroke: A substudy of the CHARISMA trial. Int J Stroke [Internet]. 2011 Feb 4 [cited 2020 Sep 17];6(1):3–9. Available from: http://journals.sagepub.com/doi/10.1111/j.1747-4949.2010.00535.x

34. Markus HS, Droste DW, Kaps M, Larrue V, Lees KR, Siebler M, et al. Dual antiplatelet therapy with clopidogrel and aspirin in symptomatic carotid stenosis evaluated using doppler embolic signal detection: The clopidogrel and aspirin for reduction of emboli in symptomatic carotid stenosis (CARESS) trial. Circulation [Internet]. 2005 May 3 [cited 2020 Sep 17];111(17):2233–40. Available from: https://www.ahajournals.org/doi/10.1161/01.CIR.0000163561.90680.1C

35. Sorensen PS, Pedersen H, Marquardsen J, Petersson H, Heltberg A, Simonsen N, et al. Acetylsalicylic acid in the prevention of stroke in patients with reversible cerebral ischemic attacks. A Danish cooperative study. Stroke [Internet]. 1983 Jan [cited 2020 Sep 17];14(1):15–22. Available from: https://www.ahajournals.org/doi/10.1161/01.STR.14.1.15

36. Uchiyama S, Sakai N, Toi S, Ezura M, Okada Y, Takagi M, et al. Final Results of Cilostazol-Aspirin Therapy against Recurrent Stroke with Intracranial Artery Stenosis (CATHARSIS). Cerebrovasc Dis Extra [Internet]. 2015 Jan 15 [cited 2020 Sep 17];5(1):1–13. Available from: https://www.karger.com/Article/FullText/369610

37. Farrell B, Godwin J, Richards S, Warlow C. The United Kingdom transient ischaemic attack (UK-TIA) aspirin trial: Final results. J Neurol Neurosurg Psychiatry [Internet]. 1951 Dec 1 [cited 2020 Sep 17];54(12):1044–54. Available from: http://jnnp.bmj.com/

38. Wang Y, Pan Y, Zhao X, Li H, Wang D, Johnston SC, et al. Clopidogrel with aspirin in acute minor stroke or transient ischemic attack (CHANCE) trial one-year outcomes. Circulation [Internet]. 2015 Jul 7 [cited 2020 Sep 17];132(1):40–6. Available from: https://www.ahajournals.org/doi/10.1161/CIRCULATIONAHA.114.014791

39. Wong KSL, Chen C, Fu J, Chang HM, Suwanwela NC, Huang YN, et al. Clopidogrel plus aspirin versus aspirin alone for reducing embolisation in patients with acute symptomatic cerebral or carotid artery stenosis (CLAIR study): a randomised, open-label, blinded-endpoint trial. Lancet Neurol [Internet]. 2010 May 1 [cited 2020 Sep 17];9(5):489–97. Available from: http://www.thelancet.com/article/S1474442210700600/fulltext

40. He F, Xia C, Zhang JH, Li XQ, Zhou ZH, Li FP, et al. Clopidogrel plus aspirin versus aspirin alone for preventing early neurological deterioration in patients with acute ischemic stroke. J Clin Neurosci [Internet]. 2015 Jan 1 [cited 2020 Sep 17];22(1):83–6. Available from: http://www.jocn-journal.com/article/S0967586814004822/fulltext

41. Yi X, Lin J, Wang C, Zhang B, Chi W. A comparative study of dual versus monoantiplatelet therapy in patients with acute large-artery atherosclerosis stroke. J Stroke Cerebrovasc Dis [Internet]. 2014 Aug 1 [cited 2020 Sep 17];23(7):1975–81. Available from: http://www.strokejournal.org/article/S1052305714000536/fulltext

42. Acheson J, Danta G, Hutchinson EC. Controlled Trial of Dipyridamole in Cerebral Vascular Disease. Br Med J [Internet]. 1969 Mar 8 [cited 2020 Sep 17];1(5644):614–5. Available from: http://www.bmj.com/

43. Fields WS. Persantine Aspirin Trial in cerebral ischemia. Part II: Endpoint results. The American-Canadian Co-Operative Study group. Stroke [Internet]. 1985 May [cited 2020 Sep 17];16(3):406–15. Available from: https://www.ahajournals.org/doi/10.1161/01.STR.16.3.406

44. Group TCCS. A Randomized Trial of Aspirin and Sulfinpyrazone in Threatened Stroke. N Engl J Med [Internet]. 1978 Jul 13 [cited 2020 Sep 17];299(2):53–9. Available from: http://www.nejm.org/doi/abs/10.1056/NEJM197807132990201

45. Fukuuchi Y, Tohgi H, Okudera T, Ikeda Y, Miyanaga Y, Uchiyama S, et al. A Randomized, Double-Blind Study Comparing the Safety and Efficacy of Clopidogrel versus Ticlopidine in Japanese Patients with Noncardioembolic Cerebral Infarction. Cerebrovasc Dis [Internet]. 2008 Feb [cited 2020 Sep 20];25(1–2):40–9. Available from: https://www.karger.com/Article/FullText/111498

46. Han SW, Lee S-S, Kim SH, Lee JH, Kim GS, Kim O-J, et al. Effect of Cilostazol in Acute Lacunar Infarction Based on Pulsatility Index of Transcranial Doppler (ECLIPse): A Multicenter, Randomized, Double-Blind, Placebo-Controlled Trial. Eur Neurol [Internet]. 2013 Feb [cited 2020 Sep 20];69(1):33–40. Available from: https://www.karger.com/Article/FullText/338247

47. Kwon SU, Hong KS, Kang DW, Park JM, Lee JH, Cho YJ, et al. Efficacy and safety of combination antiplatelet therapies in patients with symptomatic intracranial atherosclerotic stenosis. Stroke [Internet]. 2011 Oct [cited 2020 Sep 20];42(10):2883–90. Available from: https://www.ahajournals.org/doi/10.1161/STROKEAHA.110.609370

48. Britton M, Helmers C, Samuelsson K, Arbin M Von, Britton M, Carlsson A, et al. Original contributions high-dose acetylsalicylic acid after cerebral infarction. Stroke [Internet]. 1987 [cited 2020 Sep 20];18(2):325–34. Available from: http://ahajournals.org

49. Uchiyama S, Fukuuchi Y, Yamaguchi T. The safety and efficacy of clopidogrel versus ticlopidine in Japanese stroke patients: Combined results of two Phase III, multicenter, randomized clinical trials. J Neurol [Internet]. 2009 Jun 14 [cited 2020 Sep 20];256(6):888–97. Available from: https://link.springer.com/article/10.1007/s00415-009-5035-4

50. Uchiyama S, Ikeda Y, Urano Y, Horie Y, Yamaguchi T. The Japanese Aggrenox (Extended-Release Dipyridamole plus Aspirin) Stroke Prevention versus Aspirin Programme (JASAP) Study: A Randomized, Double-Blind, Controlled Trial. Cerebrovasc Dis [Internet]. 2011 May [cited 2020 Sep 20];31(6):601–13. Available from: https://www.karger.com/Article/FullText/327035

51. Shinohara Y, Nishimaru K, Sawada T, Terashi A, Handa S, Hirai S, et al. Sarpogrelate-aspirin comparative clinical study for efficacy and safety in secondary prevention of cerebral infarction (S-ACCESS): A randomized, double-blind, aspirin-controlled trial. Stroke [Internet]. 2008 Jun 1 [cited 2020 Sep 20];39(6):1827–33. Available from: https://www.ahajournals.org/doi/10.1161/STROKEAHA.107.505131

52. Candelise L, Landi G, Perrone P, Bracchi M, Brambilla G. A randomized trial of aspirin and sulfinpyrazone in patients with TIA. Stroke [Internet]. 1982 Mar [cited 2020 Sep 20];13(2):175–9. Available from: https://www.ahajournals.org/doi/10.1161/01.STR.13.2.175

53. Olsson JE, Brechter C, Bäcklund H, Krook H, Müller R, Nitelius E, et al. Anticoagulant vs anti-platelet therapy as prophylactic against cerebral infarction in transient ischemic attacks. Stroke [Internet]. 1980 Jan [cited 2020 Sep 20];11(1):4–9. Available from: https://www.ahajournals.org/doi/10.1161/01.STR.11.1.4

54. Bath PMW, Lindenstrom E, Boysen G, De Deyn P, Friis P, Leys D, et al. Tinzaparin in acute ischaemic stroke (TAIST): A randomised aspirin-controlled trial. Lancet [Internet]. 2001 Sep 1 [cited 2020 Sep 20];358(9283):702–10. Available from: http://www.thelancet.com/article/S0140673601058378/fulltext

55. Culebras A, Rotta-Escalante R, Vila J, Domínguez R, Abiusi G, Famulari A, et al. Triflusal vs aspirin for prevention of cerebral infarction: A randomized stroke study. Neurology [Internet]. 2004 Apr 13 [cited 2020 Sep 20];62(7):1073–80. Available from: https://n.neurology.org/content/62/7/1073

56. Diener H-C, Sacco RL, Easton JD, Granger CB, Bernstein RA, Uchiyama S, et al. Dabigatran for Prevention of Stroke after Embolic Stroke of Undetermined Source. N Engl J Med [Internet]. 2019 May 16 [cited 2020 Sep 20];380(20):1906–17. Available from: http://www.nejm.org/doi/10.1056/NEJMoa1813959

57. Hart RG, Sharma M, Mundl H, Kasner SE, Bangdiwala SI, Berkowitz SD, et al. Rivaroxaban for Stroke Prevention after Embolic Stroke of Undetermined Source. N Engl J Med [Internet]. 2018 Jun 7 [cited 2020 Sep 20];378(23):2191–201. Available from: http://www.nejm.org/doi/10.1056/NEJMoa1802686

58. ITO E, TAKAHASHI A, YAMAMOTO H, KUZUHARA S, UCHIYAMA S, NAKAJIMA M. Ticlopidine Alone Versus Ticlopidine Plus Aspirin for Preventing Recurrent Stroke. Intern Med [Internet]. 2003 Sep 1 [cited 2020 Sep 20];42(9):793–9. Available from: http://joi.jlc.jst.go.jp/JST.Journalarchive/internalmedicine1992/42.793?from=CrossRef
